# Orthohantavirus diversity in Central-East Argentina: Insights from complete genomic sequencing on phylogenetics, Geographic patterns and transmission scenarios

**DOI:** 10.1371/journal.pntd.0012465

**Published:** 2024-10-09

**Authors:** Daniel Oscar Alonso, Sebastián Dario Kehl, Rocío María Coelho, Natalia Periolo, Tomás Poklépovich Caride, Julián Sanchez Loria, Facundo Gabriel Cuba, Unai Pérez-Sautu, Mariano Sanchez-Lockhart, Gustavo Palacios, Carla Maria Bellomo, Valeria Paula Martinez

**Affiliations:** 1 Laboratorio Nacional de Referencia de Hantavirus, Instituto Nacional de Enfermedades Infecciosas, Administración Nacional de Laboratorios e Institutos de Salud “Dr. Carlos G. Malbran”, Ciudad Autónoma de Buenos Aires, Argentina; 2 Unidad Operativa Centro Nacional de Genómica y Bioinformática, Administración Nacional de Laboratorios e Institutos de Salud “Dr. Carlos G. Malbrán”, Ciudad Autónoma de Buenos Aires, Argentina; 3 Center for Genome Sciences, Molecular Biology Division, United States Army Medical Research Institute of Infectious Diseases, Fort Detrick, Frederick, Maryland, United States of America; 4 Department of Microbiology, Icahn School of Medicine at Mount Sinai, New York, New York, United States of America; Medizinische Universitat Wien, AUSTRIA

## Abstract

Hantavirus Pulmonary Syndrome (HPS), characterized by its high fatality rate, poses a significant public health concern in Argentina due to the increasing evidence of person-to-person transmission of Andes virus. Several orthohantaviruses were described in the country, but their phylogenetic relationships were inferred from partial genomic sequences. The objectives of this work were to assess the viral diversity of the most prevalent orthohantaviruses associated with HPS cases in the Central-East (CE) region of Argentina, elucidate the geographic patterns of distribution of each variant and reconstruct comprehensive phylogenetic relationships utilizing complete genomic sequencing. To accomplish this, a detailed analysis was conducted of the geographic distribution of reported cases within the most impacted province of the region. A representative sample of cases was then selected to generate a geographic map illustrating the distribution of viral variants. Complete viral genomes were obtained from HPS cases reported in the region, including some from epidemiologically linked cases. The phylogenetic analysis based on complete genomes defined two separate clades in Argentina: Andes virus in the Southwestern region and Andes-like viruses in other parts of the country. In the CE region, Buenos Aires virus and Lechiguanas virus clearly segregate in two subclades. Complete genomes were useful to distinguish person-to-person transmission from environmental co-exposure to rodent population. This study enhances the understanding of the genetic diversity, geographical spread, and transmission dynamics of orthohantaviruses in Central Argentina and prompt to consider the inclusion of Buenos Aires virus and Lechiguanas virus in the species *Orthohantavirus andesense*, as named viruses.

## Author summary

The discovery of hundreds of novel viruses assignable to the order *Bunyavirales* in recent years led to International Committee on Taxonomy of Viruses (ICTV) to impose the requirement of complete or almost complete viral sequences in order to establish a correct taxonomic classification. As a large number of incomplete sequences has confounded the understanding of Hantavirus diversity, many viruses have been recently declassified including several variants circulating in Argentina. In this work, high quality and complete genomic sequences were obtained of Buenos Aires and Lechiguanas viruses, which are responsible for the majority of the HPS cases in the CE region of Argentina. The information provided in this work will help to consider the re-classification of Lechiguanas and the inclusion of Buenos Aires as named viruses within the species *Orthohantavirus andesense*.

## Introduction

Hantavirus pulmonary syndrome (HPS) is a severe zoonotic disease endemic in The Americas, where it shows low incidence but high lethality. Many New World hantaviruses (NWH) have been described and associated with the disease in all the continent [1]. It is mainly associated with environmental exposure to rodents in rural and wild settings. The infection occurs by inhalation of contaminated aerosols generated by infected rodents that act as reservoirs in nature. Hantaviruses are enveloped, single-strand RNA viruses with tripartite genome consisting of small (S), medium (M), and large (L) segments [2], which encode for a nucleoprotein, a glycoprotein precursor and a RNA dependent-RNA polymerase, respectively. Pathogenic hantaviruses are currently grouped under the genus *Orthohantavirus*, family *Hantaviridae*. In South America, only five species of orthohantavirus have been recognized by the International Committee on Taxonomy of Viruses (ICTV) despite the fact that 25 distinct viruses were described, most of which have partial genetic information [3].

Andes virus (ANDV) was the first orthohantavirus identified as an etiologic agent of HPS in Argentina [4]. It was associated with up to 50% case fatality rate and person-to-person transmission outbreaks [5–13] and considered a global threat to public health. After the description of ANDV and its rodent reservoir, *Oligoryzomys longicaudatus*, many orthohantavirus variants were identified in other parts of the country [14–17]. Several of them were considered as different viruses based on incomplete genetic information or because they were identified from a different host species; however, the classification of rodent species in the genus *Oligoryzomys*, is still controversial. As several orthohantaviruses identified in Argentina are closely related to ANDV, hereafter referred to as AND-like orthohantaviruses, there is a need to understand the genetic relatedness among them to gain insight into their biological properties. ANDV and AND-like orthohantaviruses were classified under the species *Orthohantavirus andesense*.

ANDV is restricted to Southwestern Argentina and Chile [18], while AND-like orthohantaviruses were characterized from central east (CE), northwest and northeast regions of Argentina and surrounding countries [16,19–21]. Given the difficulty to be isolated, the classification of hantavirids was mostly based on genetic relatedness in partial genomic fragments [22] and the use of non-overlapping fragments could lead to the misidentification of new viruses. Until now, complete genomes were obtained only for ANDV. For AND-like orthohantaviruses, only few S- and M-segments are available. The absence of L-segment information in hantaviral taxonomic analyses is problematic because it encodes, by far, the longest protein of the genome [22]. Viral genetic variability and their genetic relatedness among members of the species remain inconclusive. Among HPS cases, the most prevalent are Orán virus (ORNV), Buenos Aires virus (BAV) and Lechiguanas virus (LECV). Regarding remarkable biological properties, BAV is particularly of great concern due to its implication in several outbreaks and suspicion of person-to-person transmission [23–25].

The objective of this work was to evaluate the viral divergence in the CE region of Argentina and reconstruct the phylogenetic relationships using complete genomic sequences. For this, the aim was to obtain complete genomes from clinical samples of HPS cases reported in the CE region.

## Methods

A retrospective and transversal study of case distribution from 1995 to 2022 was performed in the CE region including all the cases reported in the region (https://sisa.msal.gov.ar/sisa/). Samples used were available at the National Reference Laboratory for Hantavirus and the epidemiological information was obtained from the associated clinical/epidemiological form. Human samples were anonymized. In particular, Buenos Aires, the most affected province of the CE region, was selected to construct a map showing the geographical distribution of HPS cases and viral variants. Buenos Aires province surface area is 307,571 km^2^ [26] and it is divided into 135 localities. Confirmed cases without recent history travel were selected (n = 528). The distribution of cases and viral variants were mapped using QGIS software (3.16). For the construction of the variant distribution map, a sample of 98 HPS cases were selected. Partial fragments of viral genomes were sequenced to determine the variant as previously described [27].

In order to obtain complete genomes of the three variants circulating in the area, cases that were previously characterized by partial viral sequencing, were selected: 16 BAV, four LECV, and one Plata virus (PLAV). Most of BAV cases were selected due to their involvement in clusters of epidemiologically related cases, which originated the suspicion of person-to-person transmission between them and were reported previously[23,24], while others were temporally distant but reported in the same locations. The selection of three LECV cases and one rodent was based on their distant geographic origin, to estimate the highest genetic distance. The case of PLAV, was selected by the availability of the sample. For the genetic analysis, RNA was extracted from 400 μl of whole blood in TRIzol LS reagent using the TRIzol Reagent and Phasemaker Tubes Complete System (ThermoFisher Scientific, Waltham, MA, USA) following the manufacturer’s instructions. For whole viral genome sequencing, libraries were prepared by bait and amplicon strategies for viral RNA enrichment. Most of the S- and M-segments were obtained by bait enrichment as previously described [10] An amplicon based method was implemented for the L-segment and for those cases from which full S- and M-segments were not successfully obtained by bait enrichment technique (S1 Fig). This strategy was design for the amplification of one or two overlapping fragments by a RT-PCR step (Superscript IV One step RT-PCR system, Invitrogen) followed, if necessary, by a heminested PCR (Hi Fidelity Taq Platinum, Invitrogen). All end-point PCR reactions were carried out in an Applied Biosystems 2720 Thermal Cycler. The list of primers for amplification is available upon request. A bead based method was used for purification of amplicon PCR products (Illumina tune beads). Pooled libraries were sequenced on the Illumina MiSeq, NextSeq or NovaSeq sequencing platforms (Illumina, San Diego, CA). Bioinformatic analysis on fastQ resulting files were performed as previously described Martinez et al 2020 [10] supplementary material. To generate BAV and LECV consensus L-segments, cleaned reads were assembled *de novo* using SPAdes v3.9.0.5; then, these *de novo* assembled sequences were used as reference to align clean reads with Bowtie 2.4. Only bases with a Phred quality score >Q30 and a minimum of 10X coverage were used for consensus calling. Consensus genome sequences from cases were aligned using MAFFT v.7.397. The phylogenetic trees were constructed using IQTREE with ModelFinder for model selection, and ultrafast bootstrap analysis with 1000 replicates. The best-fit model according to BIC was GTR+F+I+G4, and this model was used for tree reconstruction. Bootstrap support was based on 1,000 maximum-likelihood replicates.

The diversity of the viral population was then estimated according to two parameters: the total number of individual nucleotide changes in each genomic sequence and the total number of amino acid changes in the coding regions of each segment. The percentage of divergence was determined by alignment analysis with the basic local alignment search tool, BLAST [28]. BlastN was selected for the comparison of more dissimilar sequences. The remaining parameters were set by default.

## Results

**Fig 1:**
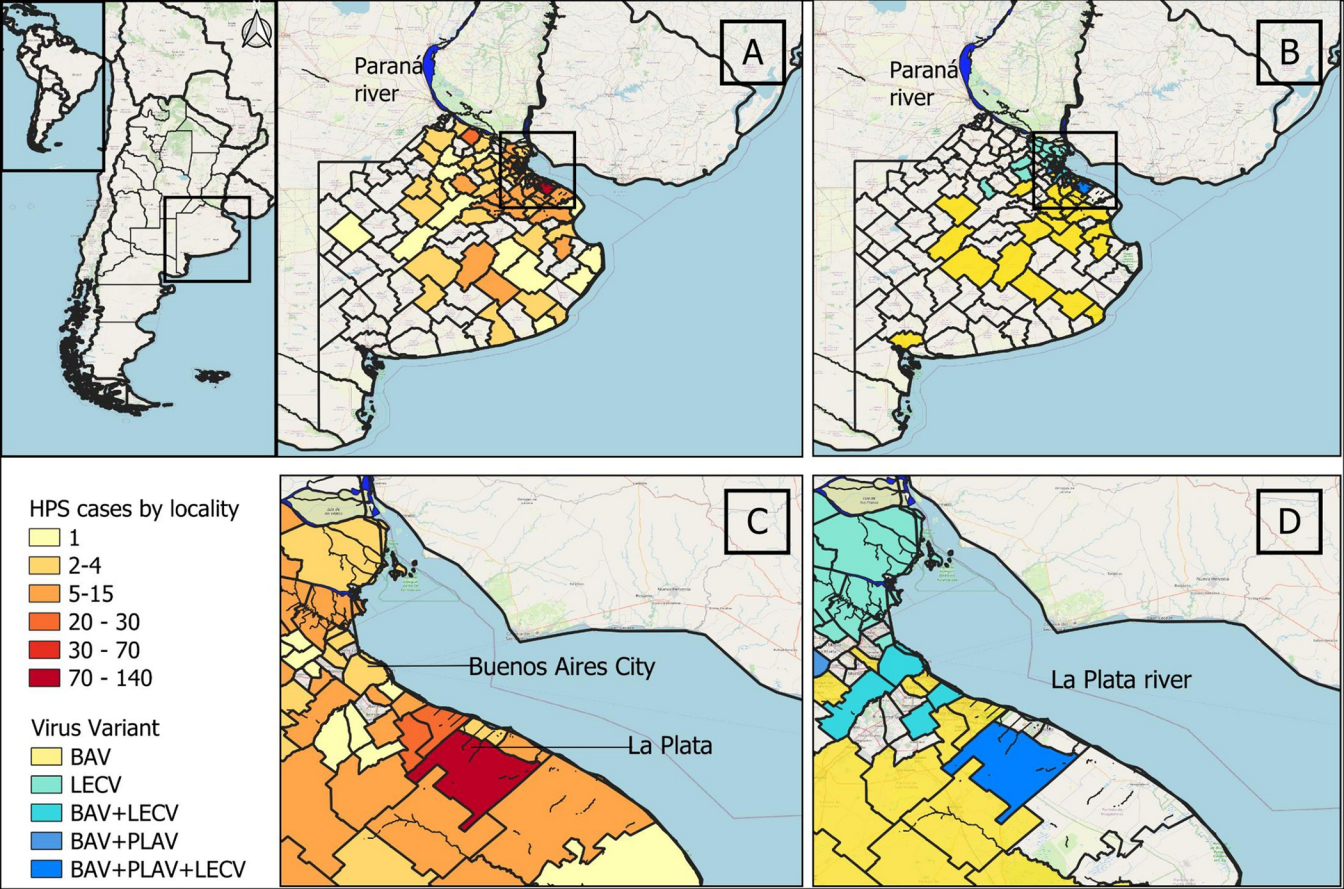
Geographic distribution of hantavirus pulmonary syndrome in Buenos Aires province, Argentina. A: distribution of HPS cases reported by localities in the province during the period 1995–2022 (n = 528). B: distribution of viral variants by localities (n = 98). C and D showed the amplification of the areas in the squares of A and B respectively. Geospatial data was obtained by using the QGIS XYZ plugin, accessing data provided by OpenStreetMap (OpenStreetMap contributors, 2024). OpenStreetMap contributors. "OpenStreetMap." 2024. Web. https://www.openstreetmap.org -"America".- The vector data of the provinces and municipalities of Argentina were obtained from the National Geographic Institute: https://www.ign.gob.ar/NuestrasActividades/InformacionGeoespacial/CapasSIG.

The CE endemic region of HPS comprises parts of three provinces. The number of reported cases in the region during the period 1996–2022 was 934. Buenos Aires province (BAP) was the most affected according to the number of accumulated cases (n = 678, 72.6%). The distribution of 528 cases was studied within localities. The distribution of cases in the province was wide, with highest records in localities placed near riverside areas with shores of the La Plata and Paraná rivers, and other minor rivers that flow into the Atlantic Ocean (Fig 1A). From 135 localities, 117 (86.7%) reported at least one HPS case, where the number of cases per locality varied from 1 to 134. The most affected were rural areas around La Plata and surrounding localities (Fig 1C). Among the cases, 98 were selected for virus characterization, 66.3% were associated with BAV, 26.5% with LEV and 7.1% with PLAV. The pattern of geographic distribution of each virus was different (Fig 1B). LECV was more frequently found in the northern border of Buenos Aires city along La Plata river through Paraná river, while BAV was widely distributed in the rest of the province from the Delta of La Plata river to the south and southwest. PLAV was found sporadically. The three variants were found cocirculating in the surrounding localities of La Plata (Fig 1D).

**Fig 2:**
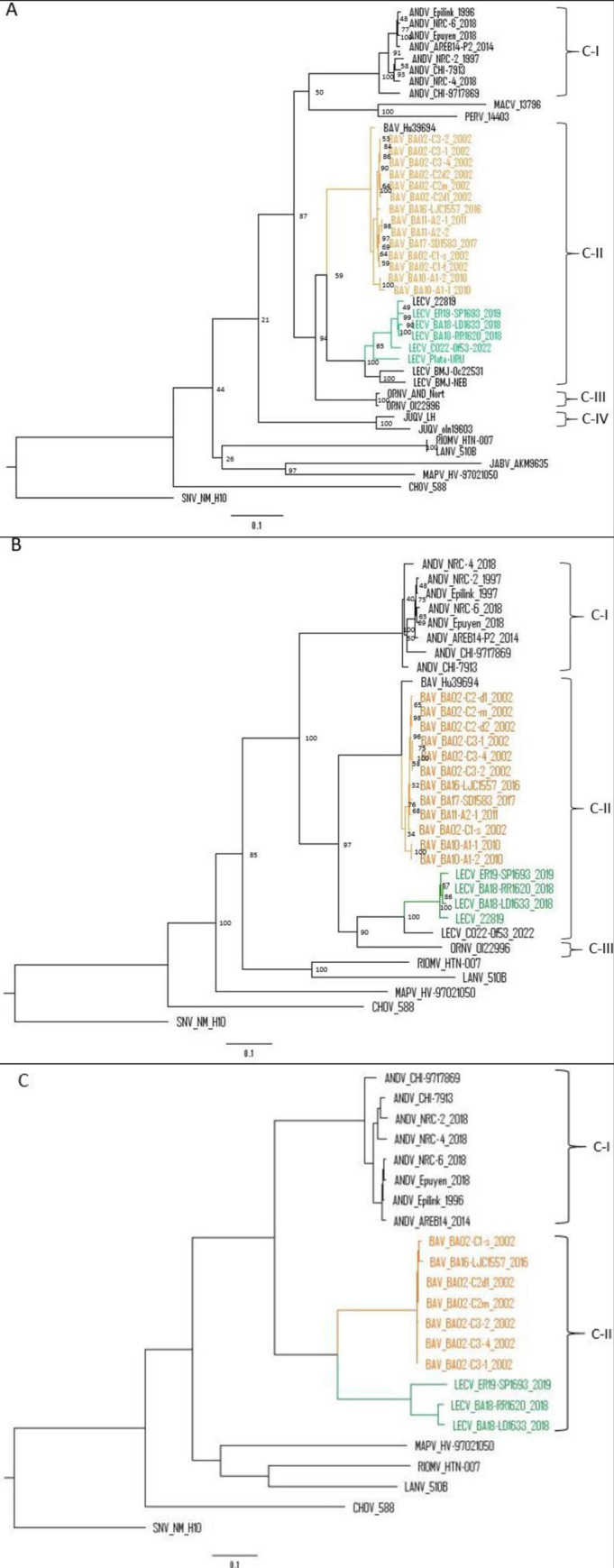
Maximum likelihood phylogenetic analysis based on complete genomes of orthohantaviruses in Argentina. A: S-segment; B: M-segment; and C: L-segment. Orange letters and lines represent BAV, green letters and lines represent LECV. Square brackets show number of clusters.

Nine complete (S-, M- and L- segments) and eight incomplete genomes (complete S- and/or M-segments) were obtained (Table 1). Additionally, an almost complete sequence with only 60.4% of coverage in the L-segment was obtained. Phylogenetic analysis was performed together with all the available complete sequences in GenBank identified in Argentina, including ANDV and other NWH (Fig 2). The analysis of M- and L- segments showed two main clades which clearly segregate ANDV (C-I) -distributed only in southwestern Argentina- from AND-like orthohantaviruses. Clade II groups variants from CE region (C-II) and clade III is represented by ORNV, from the northwest region (C-III, only in the M-segment tree). Particularly, in the tree of the S-segment, as there are more complete sequences available, the phylogenetic reconstruction revealed two branches inside C-II represented by BAV and LECV; PLAV and other viruses previously described outside the study area (Neembucú and Bermejo)[29,30] grouped together with LECV. In the same tree, other pathogenic viruses were clearly separated in well-defined clades as the pathogenic ORNV and Juquitiba virus (JUQV) (C-III and V, respectively).

**Table 1:** Cases of hantavirus pulmonary syndrome selected for sequencing.

ID	Cluster	Case	Age	Date of onset	Residence	Probable place of infection or risk activity	Sequence coverage
1	C1	C1-f	41	7/10/2002	CABA, urban area	Farm in LP, rural area	S complete
2		C1-s	14	8/8/2002	CABA, urban area	Shared the weekend at his father´s house (13 to 14th July/02)	S, M & L complete
3	C2	C2-d1	12	7/24/2002	LP-El Peligro, rural area (BAP)	LP-El Peligro, rural area (BAP)	S & M complete; L 60.4%
4		C2-d2	11	7/28/2002			S, M & L complete
5		C2-s	*NA*	*NA*			*NA*
6		C2-m	40	8/4/2002			S, M & L complete
7	C3	C3-1	28	8/26/2002	LP-Abasto, rural area (BAP).	LP-Abasto, rural area (BAP).	S, M & L complete
8		C3-2	27	8/29/2002			S, M & L complete
9		C3-3	21	9/10/2002			*NA*
10		C3-4	30	9/14/2002			S, M & L complete
11	A1	A1-1	54	3/4/2010	CABA, urban area	CA (BAP)	S complete
12		A1-2	52	3/30/2010	SAP, rural area (BAP)	Contact with A1-1 (husband)	S & M complete
13	A2	A2-1	58	9/5/2011	BZ, urban area (BAP)	FV, suburban area (BAP)	S complete; M 82%
14		A2-2	67	9/29/2011	BZ, urban area (BAP)	Contact with A2-1 (husband)	S complete
15		LJC1557	29	12/5/2016	LP-Abasto, rural area (BAP).	LP-Abasto, rural area (BAP).	S, M & L complete
16		SD1583	30	09/30/2017	LP-El Peligro, rural area (BAP).	LP-El Peligro, rural area (BAP).	S complete; M 91.2%
17		SP1693	37	12/24/2018	Gualeguay, rural area (ERP)	Gualeguay, rural worker	S, M & L complete
18		LD1633	49	3/6/2018	CABA, urban area	Alberti (BAP), fishing & camping	S, M & L complete
19		RR1620	38	1/16/2018	Don Torcuato (BAP)	Unknown	S, M & L complete
20		URU_1	NA	1997	Uy	Unknown	S complete

BZ: Berazategui; BAP: Buenos Aires province; CABA = Buenos Aires city; CA: Costa Atlántica; ERP: Entre Rios province; FV: Florencio Varela; LP = La Plata; SAP: San Antonio de Padua; Uy: Uruguay. NA = not available.

For viral diversity analysis in our dataset, we first estimated the overall genetic variability with the complete genomes (S-, M- and L-segments) of each branch of Clade II. The nucleotide divergence range was 0.6–1.2% (n = 4) for BAV and 1.8–6.7% (n = 3) for LECV. Then, we estimated the divergence between representative viruses of each phylogenetic clade or branch (Table 2). Compared with ANDV, BAV and LECV diverged 20 and 20.7% respectively, while BAV compared with LECV showed 17.7% of divergence at nucleotide level. Considering the three segments separately, the divergence at the nucleotide level was similar between segments. However, at the amino acid level the divergence was higher in the M segment: ANDV vs. BAV: 8%; ANDV vs LECV: 7%; and ANDV vs. ORNV: 6%. The divergence in amino acid was remarkably lower for the S segment, indicating a high degree of conservation among all variants present in the country. On the contrary, the S segment non-coding region was the most divergent part of the genome among the different viruses mainly due to specific patterns of insertions and deletions (S2 Fig).

**Table 2:** Nucleotide and amino acid comparison between pathogenic orthohantaviruses from Argentina.

	Complete genomes												Partial genomes				
	Andes				Buenos Aires				Lechiguanas				Orán		Plata	Bermejo	Juquitiba
	MN258239	MN258205	MN258172		OR908884	OR965909	PP151166		OR908897	PP151163	PP003836		AF482715	AF028024	PP504848	AF482713	KY053844
	S	M	L	S/M/L	S	M	L	S/M/L	S	M	L	S/M/L	S	M	S	S	S
ANDV	**—**				3.0	8.0	5.3	**5.2**	3.3	7.0	5.1	**5.3**	3.5	6.0	3.5	3.3	5.4
BASV	19.4	21.0	19.0	20.0	**—**				0.5	4.0	2.8	**2.6**	0.9	6.0	0.5	0.2	4.9
LECV	21.4	21.0	19.9	20.7	17.1	18.0	18.0	17.7	**—**				1.4	6.0	0.2	0.0	5.2
ORNV	21.0	21.0	~	~	16.6	19.0	~	~	16.1	19.0	~	~	**—**		1.6	1.4	4.4
PLAV	22.0	~	~	~	15.6	~	~	~	9.2	~	~	~	14.9		**—**	0.2	5.4
BRJV	21.6	~	~	~	15.7	~	~	~	10.0	~	~	~	16.4	~	10.8	**—**	5.1
JUQV	18.2	~	~	~	19.9	~	~	~	23.6	~	~	~	16.6	~	23.3	17.4	**—**

Comparisons were done on complete sequences from the three segments separately (S, M or L) and from complete genomes (S/M/L). Values below dashes (which indicate 0% divergence) are nucleotide sequence comparisons, and those above the dashes are deduced amino acid sequence comparisons. ~ indicate not available. ANDV: Andes virus; BASV: Buenos Aires virus; LECV: Lechiguanas virus; ORNV: Orán virus; PLAV: Plata virus; BRJV: Bermejo virus; JUQV: Juqitiba virus

**Fig 3:**
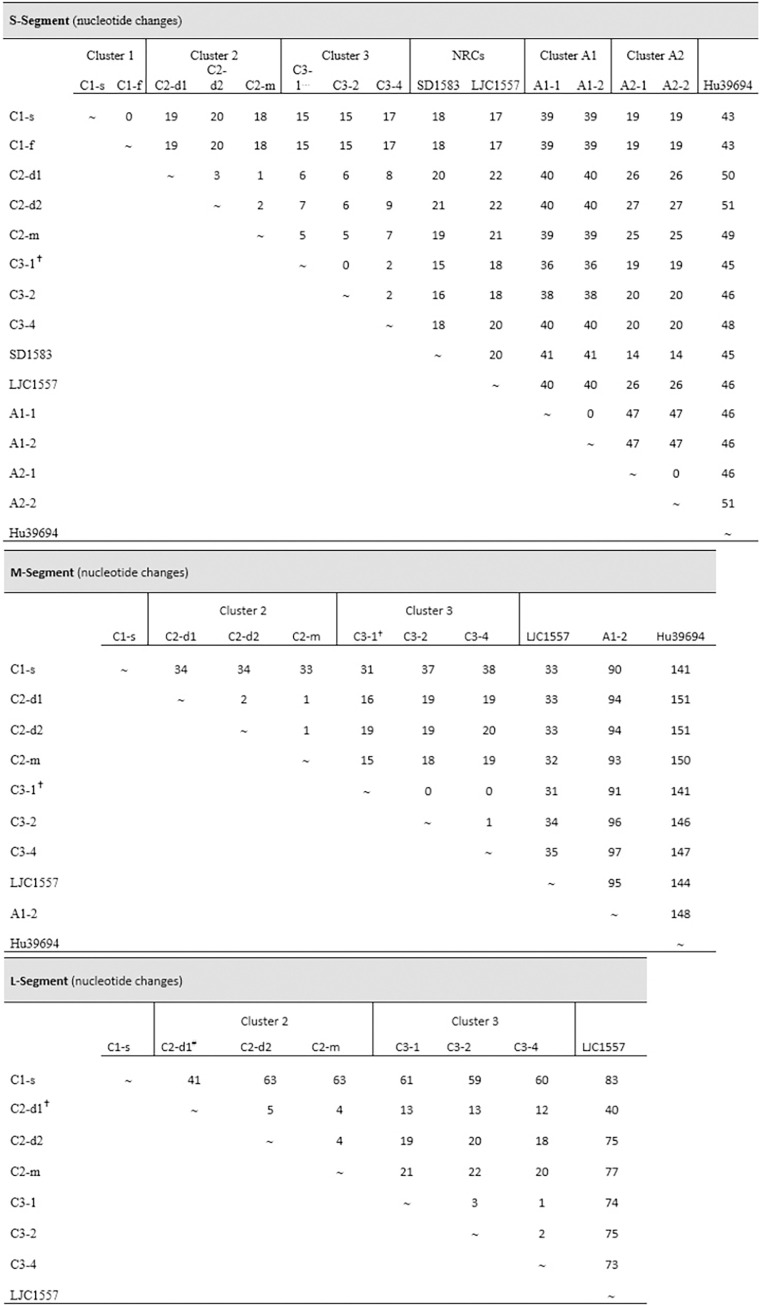
Nucleotide changes in the S, M and L segments among hantavirus pulmonary syndrome cases, both within and between clusters. Comparisons were made using available sequences. this sequence has 6 undetermined nucleotides (N) in the S-, and 168 in the M-segments; ^#^ *sequence with 60*.*4% of coverage in the L-segment*.

In previous works, epidemiologically linked HPS cases reported in BAP were analyzed, all of them associated with BAV. Despite 100% of nucleotide identity in partial fragments (total = 1000 bp from S- and M-segments) between cases in three clusters, person-to-person transmission was confirmed only in one (cluster 1) based on the epidemiological data [23]. In the present work, a deeper comparative analysis was performed with complete genomes of some of these clustered cases and with complete S-segment sequences of other cases (Table 1) [24]. In cluster 1, the complete genome of C1-s was obtained, but only the S-segment of C1-f; therefore 100% of nucleotide identity could be confirmed only in the S-segment. The same results were obtained for clusters A1 and A2, (100% nucleotide identity in the S-segment). Interestingly, in clusters C1, A1 and A2 the place of exposure for the secondary cases could not determine (Fig 3). On the contrary, comparison in cluster 2 and cluster 3 revealed changes in the whole genome. In cluster 2, where the symptom onset of all the cases occurred within a period of 11 days, the three genomes obtained showed at least one change in each segment. The most divergent was C2-d1, which differed from C2-d2 in ten residues in the whole genome. Considering that the coverage of the L-segment of C2-d1 was 60.4%, the total number of changes might be higher. Cluster 3, represents another scenario of possible coexposure in the same house with a maximum period of symptom onset of 20 days between the first and the last cases (C3-1 and C3-4). In this cluster, the differences were up to five residues between C3-2 and C3-4 (Fig 3). Intercluster comparisons were performed including non-related cases from the same locality (La Plata) but reported more than 10 years after. The comparisons showed a clear relation between genetic divergence and geographic distance (Fig 3), as evidenced by the highest divergence of Hu39694 (original from Pergamino, 285 km distant from La Plata).

## Discussion

Since the identification of the first etiologic agent of HPS in Argentina in 1996, enormous efforts have been channelled to understand viral diversity, routes of transmission and pathogenicity [5,10,11,23,39,40]. Most of the HPS causing agents in Argentina and in South America were classified under the species *Orthohantavirus andesense*. Their close relationship with ANDV is a cause for concern in the region because of its ability to spread from person-to-person and high lethality. A large number of incomplete sequences has confounded the understanding of hantavirus diversity. In this work, for the first time the complete genome of the most prevalent HPS agents in the CE region of Argentina- BAV and LECV- were reported (Table 3). Nine new complete genomes were obtained from clinical samples of HPS cases that were useful to evaluate genetic variability of each virus. Up to now, only L-segment sequences of ANDV were available. The ICTV Hantaviridae Study Group decided to reassess the entire *Hantaviridae* family using a stringent criterion which implies to assess only viruses for which there is S + M + L coding-complete or near-complete sequence information, and this resulted in the abolishment of some orthohantavirus species and the declassification (removal from established species) of an additional several orthohantaviruses, including LECV and ORNV [22]. The information provided in this work will help to consider the re-classification of LECV, and the inclusion of BAV as named viruses within *Orthohantavirus andesense*.

**Table 3:** List of sequences and strains utilized in the comparative genomics study of American orthohantaviruses.

Virus name	Source	Strain name	Location	Year	GenBank Acc. Nª			Reference	Denomination in Fig 2
					S	M	L		
ANDV	Human	NRC-4_2018	Villa Meliquina, Ne, AR	2018	MN258226	MN258192	MN258159	Martinez *et al*. [10]	ANDV_NRC-4_2018
ANDV	Human	ARG-Epuyen	Epuyén, Ch, AR	2018	MN258239	MN258205	MN258172	Martinez *et al*. [10]	ANDV_Epuyén_2018
ANDV	Human	NRC-6_18	El Hoyo, Ch, AR	2018	MN258228	MN258194	MN258161	Martinez *et al*. [10]	ANDV_NRC-6_2018
ANDV	Human	ARG-Epilink	El Bolsón, RN, AR	1996	MN258223	MN258189	MN258156	Martinez *et al*. [10]	ANDV_Epilink_1996
ANDV	Human	NRC-2_1997	Bariloche, RN, AR	1997	MN258224	MN258190	MN258157	Martinez *et al*. [10]	ANDV_NRC-2_2018
ANDV	Human	AREB14-P2	El Bolsón, RN, AR	2014	MN850084	MN850089	MN850094	Alonso et al. [11]	ANDV_AREB14-P2_2014
ANDV	*O*. *longicaudatus*	CHI-9717869	Coyhaique, Aysén, Chile	1997	MT956622	MT956623	MT956621	Warner *et al*.[31]	ANDV/CHI-9717869
ANDV	Human	CHI-7913	Mulchén, Biobío, Chile	1999	MT956618	MT956619	MT956620	Warner *et al*.[31]	ANDV/CHI-7913
BAV	Human	Hu39694	Pergamino, BA, AR	NA	AF482711	AF028023	NA	Bohlman et al[30]	BAV/Hu39694
BAV	Human	BA02-C1f	La Plata, BA, AR	2002	PP504847	NA	NA	Bellomo *et al*. [17]	BAV_BA02-C1-f_2002
BAV	Human	BA02-C1s	La Plata, BA, AR	2002	OP555730	OP555731	PP151165	Bellomo *et al*. [17] And This work	BAV_BA02-C1-s_2002
BAV	Human	BA02-C2d1	La Plata, BA, AR	2002	OR908884	OR965909	PP151166	**This work**	BAV_BA02-C2-d1_2002
BAV	Human	BA02-C2d2	La Plata, BA, AR	2002	OR908885	OR965910	PP504849	**This work**	BAV_BA02-C2-d2_2002
BAV	Human	BA02-C2m	La Plata, BA, AR	2002	OR908887	OR965911	PP151167	**This work**	BAV_BA02-C2-m_2002
BAV	Human	BA02-C3-1	La Plata, BA, AR	2002	OR908886	OR987850	PP151168	**This work**	BAV_BA02-C3-1_2002
BAV	Human	BA02-C3-2	La Plata, BA, AR	2002	OR908888	OR987851	PP151169	**This work**	BAV_BA02-C3-2_2002
BAV	Human	BA02-C3-4	La Plata, BA, AR	2002	OR908889	OR987852	PP151170	**This work**	BAV_BA02-C3-4_2002
BAV	Human	BA16-LJC1557	La Plata, BA, AR	2016	OR908890	OR987853	PP151171	**This work**	BAV_BA16-LJC1557_2016
BAV	Human	BA17-SD1583	La Plata, BA, AR	2017	OR908891	PP003838	NA	**This work**	BAV_BA17-SD1583_2017
BAV	Human	BA10-A1-1	Costa Atlántica, BA, AR	2010	OR908892	OR987854	NA	**This work**	BAV_BA10-A1-1_2010
BAV	Human	BA10-A1-2	Costa Atlántica, BA, AR	2010	OR908893	OR987855	NA	**This work**	BAV_BA10- A1-2_2010
BAV	Human	BA11-A2-1	Berazategui, BA, AR	2011	OR908894	PP003839	NA	**This work**	BAV_BA11- A2-1_2011
BAV	Human	BA11-A2-2	Berazategui, BA, AR	2011	OR908895	NA	NA	**This work**	BAV_BA11-A2-2_2011
ORNV	*O*. chacoensis*	Ol22996	Orán, Sa, AR	NA	AF482715	AF028024	NA	Levis *et al*. [19]	ORNV_Ol22996
ORNV	*O*. *chacoensis*	AND Nort	Orán, Sa, AR	1997	AF325966	NA	NA	Gonzalez Della Valle *et al*.[32]	ORNV_AND Nort_1997
LECV	*O*. *chacoensis*	BMJ-ÑEB	Ñeembucu, PY	NA	DQ345763	NA	NA	Chu,Y *et al*.[29]	LECV_BMJ-NEB
LECV	*O*. *flavescens*	22819	Lechiguanas islands ER, AR	NA	AF482714	AF028022	NA	Bohlman et al[30] & Levis *et al*. [19]	LECV_22819
LECV	Human	ER19-SP1693	Gualeguaychú, ER, AR	2019	OR908896	OR987856	PP151172	**This work**	LECV_ER19-SP1693_2019
LECV	Human	BA18-RR1620	Don Torcuato, BA, AR	2018	OR908897	PP151163	PP003836	**This work**	LECV_BA18-RR1620_2018
LECV	Human	BA18-LD1633	Alberti, BA, AR	2018	OR908898	PP151164	PP003837	**This work**	LECV_BA18-LD1633_2018
LECV	*O*. *flavescens*	CO-Of53	Parque Nacional Iberá, Co, AR	2023	OR890439	OR890440	NA	**This work**	LECV_CO22-Of53_2022
LECV	Human	Plata-URU	Uruguay	NA	PP504848	NA	NA	**This work**	LECV_Plata-URU
LECV	*O*. *sp*.	BMJ-Oc22531	Orán, SA, AR	NA	AF482713	NA	NA	Bohlman et al[30]	LECV-BMJ_Oc22531
JUQV	*O*. *nigripes*	Oln19603	Rio Claro, Rio de Janeiro, BZ	2015	KY053844	NA	NA	Oliveira R *et al*. [21]	JUUQV_Brazil_On19603_2015
JUQV	*Human*	LH_076_12	Santa Catarina, BZ	NA	JX173798	NA	NA		JUQV_Brazil_LH_076_12
JABV	*Akodon montensis*	Akm9635	NA, Santa Catarina, BZ	2006	JN232078	NA	NA	Oliveira R *et al*. [21]	JABV_Akm9635_2006
PERV	*Akodon azarae*	14403	Pergamino, BA, AR	1997	AF482717	NA	NA	Bohlman et al[30]	PRG_Aa14403
MACV	*Necromys benefactus*	13796	Maciel, SF, AR	1997	AF482716	NA	NA	Bohlman et al[30]	MACV_Nb13796
MAPV	*O*. *fulvescens*	HV-97021050	Western Venezuela, VE	2004	AY267347	AY363179	EU788002	Fulhorst *et al*.[33]	MAPV_HV-97021050
RIOMV	*O*. *microtis*	HTN-007	Iquitos, Maynas, PE	2010	FJ532244	FJ608550	FJ809772	Richter *et al*.[34]	RIOMV_HTN-007
LNV	*Calomys laucha*	510B	Chaco, PY	1997	AF005727	AF005728	NA	Johnson *et al*.[35]	LANV_510B
LNV	*Calomys laucha*	H731172/BRA259	Nova Olímpia, Paraná, BZ	2007	NA	NA	JX443696	Firth *et al*.[36]	LANV_H731172_BRA259
SNV	Human	NM H10	Four Corners Area, New Mexico, USA	1994	L25784	L25783	L37901	Spiropoulou et al. [37]	SNV_NM H10
CHOV	*O*. *fulvescens*	588	Panamá	2015	KT983771	KT983772	EF397003	Nelson *et al*.[38]	CHOV_588

O.: Oligoryzomys

* originaly reported as *O*. *longicaudatus*, however this species is not present in northern Argentina. ANDV = Andes virus, BAV = Buenos Aires virus, CHOV: Choclo virus; JABV: Jaborá virus; JUQV = Juquitiba virus; LECV = Lechiguanas virus, LNV = Laguna Negra virus, MACV: Maciel virus; MAPV: Maporal virus; ORNV: Orán virus; PERV: Pergamino virus; RIOMV: Río Mamoré virus; SNV = Sin Nombre virus, NE = Neuquén, Ch = Chubut, RN = Rio Negro, BA = Buenos Aires, Sa = Salta, PY = Paraguay, ER = Entre Rios, Co = Corrientes, AR = Argentina, BZ = Brazil, VE = Venezuela, PE = Peru, USA = United States of America.

The phylogenetic analysis together with complete genomes from Argentina showed two main clades with all the sequences of ANDV grouped in Clade I (C-I) and those from the CE region in Clade II (C-II). Inside C-II, BAV and LECV are defined in two branches. BAV and LECV differed from ANDV only in 5.2 and 5.3% in amino acids respectively. PLAV, Bermejo and Neembucú grouped in the subclade together with LECV and differed between them in up to 11% and 0.2% in nucleotides and amino acids respectively; then, all these variants should be considered strains of LECV. However, for the definite classification of these variants, complete sequences should be obtained.

It was estimated that almost 70% of HPS cases in BAP were caused by BAV, which has a wider geographic distribution than LECV in the province. Nonetheless, while BAV is restricted to BAP, LECV showed a wider distribution outside BAP to the north (even in the northeast region of Argentina) and to the east (Uruguay) [16,41,42]. Despite the distribution showed in this work, two cases of BAV were previously reported in the northwest region, evidencing the need to address viral genotyping studies in the whole country [27,43]. The distinctive geographic distribution pattern of BAV and LECV are probable indicators of favourable ecological conditions for different reservoir hosts.

Despite that BAV and LECV showed similar levels of divergence from ANDV, only BAV was implicated in person-to-person transmission and in several clustered cases as well [6,23–25,44]. In previous works 100% nucleotide identity was found in partial fragments of viral genomes (around 10% of the genome) in three clusters of epidemiologically linked cases. Person-to-person transmission between clustered cases was postulated based on accurate epidemiologic information that probed that the secondary cases had not been exposed to rodents or, at least, not to the same rodent population [23,24]. In the present study, 100% of nucleotide identity was found in the S-segment in three clusters, adding genetic evidence in favour of person-to-person transmission of previous publications. In contrast, several nucleotide changes were found when comparing complete genomes between cases in two clusters where co-exposure was evident. In cluster 2, person-to-person transmission should be discarded due to the number of changes found in the complete viral genomes, besides the short period of symptoms onset previously reported between all members of the family; these two considerations could indicate infections by different rodents. In cluster 3, all infections could have occurred from different rodents as well, however, person-to-person could not be ruled out between C3-1 and C3-4, because they showed only two nucleotide changes and 20 days between symptoms onset. Previous findings in the sustained ANDV person-to-person outbreak that occurred in Epuyén in 2018, showed up to two mutations in only six patients in a chain of transmissions that involved 33 cases [10]. A question that frequently arises from genomic analysis when facing clusters of cases is which would be the threshold of changes to differentiate person-to-person transmission from co-exposure to the same infected rodent. The answer remains elusive and requires deeper studies involving rodent reservoir populations. Nevertheless, the findings reported here are important and could help to resolve uncertainties in future outbreaks.

In conclusion, high quality and complete genomic sequences were obtained of many isolates of two viruses responsible for the majority of the HPS cases in the CE region of Argentina. Our results showed that both viruses diverge in 17.7% and 2.6% at nucleotide and amino acid levels respectively, show different geographical distribution patterns, and also differ in the biological property to spread from person-to-person, a mechanism only described for BAV to date. Further efforts should be focused on obtaining new complete genomes from cases and rodent host populations to fill the gaps in hantavirus classification to understand viral diversity and biological traits such as host range, routes of transmission and pathogenesis. Finally, complete genomic analysis has become a critical tool for the distinction of viral spillover from person-to-person transmissions. This study enhances our understanding of the genetic diversity, geographical spread, and transmission dynamics of orthohantaviruses involved with HPS in Central Argentina.

## Supporting information

S1 Table Coverage for each sequenced sample. (XLSX)

S1 Fig Depth dispersion and coverage by sequenced position of the L segment from a case of Buenos Aires virus, data obtained from bam files. Those positions in the genome with a depth greater than 300x are shown with green dots, while those with a depth less than this value are shown in yellow. The plotted percentage of coverage includes only those regions with a depth greaeter than 300x.
(TIF)

S2 Fig Multiple alignment detailed for the S segment. (PDF)
