# Supplementary figures and images for: Orthohantavirus diversity in Central-East Argentina: Insights from complete genomic sequencing on phylogenetics, Geographic patterns and transmission scenarios
Source: PLoS Negl Trop Dis. 2024 Oct 9;18(10):e0012465. doi: 10.1371/journal.pntd.0012465 (PMC11493241; doi:10.1371/journal.pntd.0012465)

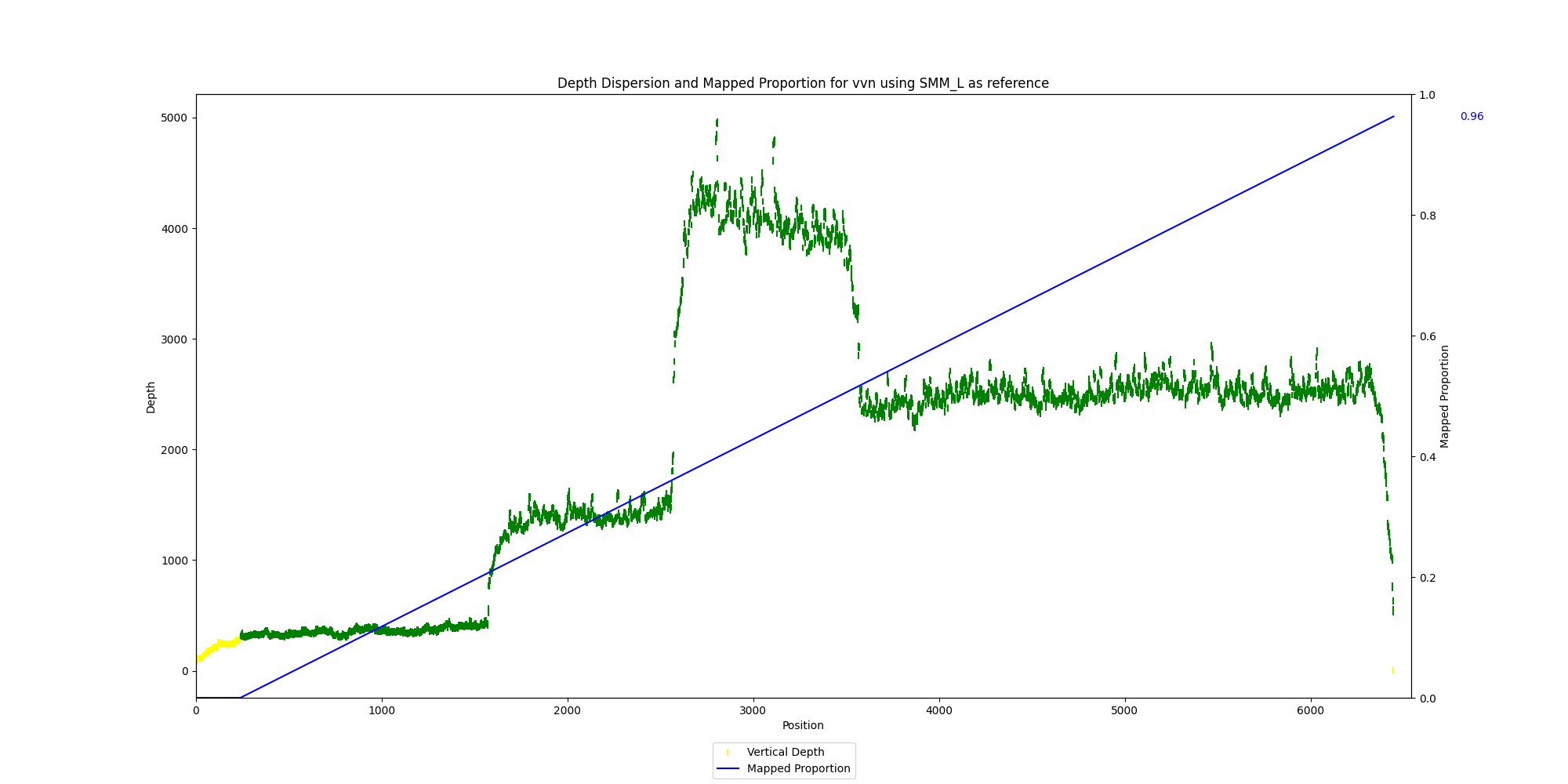

Supplement: S1 Fig — Those positions in the genome with a depth greater than 300x are shown with green dots, while those with a depth less than this value are shown in yellow. The plotted percentage of coverage includes only those regions with a depth greaeter than 300x. (TIF) [file pntd.0012465.s002.tif]
