# Supplementary material for: Orthohantavirus diversity in Central-East Argentina: Insights from complete genomic sequencing on phylogenetics, Geographic patterns and transmission scenarios
Source: PLoS Negl Trop Dis. 2024 Oct 9;18(10):e0012465. doi: 10.1371/journal.pntd.0012465 (PMC11493241; doi:10.1371/journal.pntd.0012465)
Supplement: S2 Fig — (PDF) [file pntd.0012465.s003.pdf]

```
               ....|....| ....|....| ....|....| ....|....| ....|....| ....|....|
                   5         15         25         35         45         55
SNV NM H10     tagtagtaga ctccttgaga agctactacg actaaagctg gaatgagcac cctcaaagaa
RIOMV HTN-     tagtagtaga ctccttgaga agctactatt acataagctg tgatgagcaa cctccaagaa
LANV 510B      tagtagtaga ctccttgaga agctactatt acataagctg tgatgagcaa cctccaagaa
CHOV 588       tagtagtaga ctccttgaga agctactatg actaaagctg gaatgagcac cctcaaagaa
JABV AKM96     tagtagtaga ctccttgaga agctactact acgtaagctg aaatgagcaa cctccaagac
MAPV HV-97     tagtagtaga ctccttgaga agctactgct acaaaagctg tgatgagcaa cctccaagaa
JUQV oln19     tagtagtaga ctccttgata agctactact gcaaaagctg gaatgagcaa cctccaagac
JUQV LH        tagtagtaga ctccttgata agctactact acaaaagctg gaatgagcaa cctccaagac
PERV 14403     tagtagtaga ctccttgaga agctactact gcaaaagctg gaatgagcac cctccaagaa
MACV 13796     tagtagtaga ctccttgata agctactgct gtaaaagctg gaatgagcaa cctccaagaa
ORNV O1229     tagtagtaga ctccttgaga agctactact gcgaaagctg gaatgagcac cctccaagaa
ORNV AND N     tagtagtaga ctccttgaga agctactact gcgaaagctg gaatgagcac cctccaagaa
LECV BMJ-N     --gtagtaga ctccttgaga agctactact gcgaaagctg gaatgagcac cctccaagaa
LECV BMJ-O     -agtagtaga ctccttgaga agctactact gcgaaagctg gaatgagcac cctccaagaa
LECV Plata     tagtagtaga ctccttgaga agctactgct gctaaagctg gaatgagcac cctccaagaa
LECV CO22-     tagtagtaga ctccttgaga agctactact gcgaaagctg gaatgagcac cctccaagaa
LECV BA18-     ---------- --ccttgaga agctactact gcgaaagctg gaatgagcac cctccaagaa
LECV BA18-     ---------- ---------- ---------t gcgaaagctg gaatgagcac cctccaagaa
LECV ER19-     ----agtaga ctccttgaga agctactact gcgaaagctg gaatgagcac cctccaagaa
LECV 22819     tagtagtaga ctccttgaga agctactact gcgaaagctg gaatgagcac cctccaagaa
BAV BA10-A     ---------- ---------- ---------- ---aaagctg gaatgagcac cctccaagaa
BAV BA16-L     ---------- -tccttgaga agctactact gcaaaagctg gaatgagcac cctccaagaa
BAV BA02-C     ---------- ---------- ---------- ---aaagctg gaatgagcac cctccaagaa
BAV BA17-S     ---------- ---------- ---------- ---------- --------ac cctccaagaa
BAV BA11-A     ---------- ---------- ---------- ---------- --atgagcac cctccaagaa
BAV BA02-C     -----gtaga ctccttgaga agctactact gcaaaagctg gaatgagcac cctccaagaa
BAV BA02-C     ----agtaga ctccttgaga agctactact gcaaaagctg gaatgagcac cctccaagaa
BAV BA02-C     ---------- ---cttgaga agctactact gcaaaagctg gaatgagcac cctccaagaa
BAV Hu3969     ---tagtaga ctccttgaga agctactact gcaaaagctg gaatgagcac cctccaagaa
ANDV CHI-9     cagtagtaga ctccttgaga agctactgct gcgaaagctg gaatgagcac cctccaagaa
ANDV AREB1     tagtagtaga ctccttgaga agctactgct gcgaaagctg gaatgagcac cctccaagaa
ANDV Epuye     ---------- ctccttgaga agctactgct gcgaaagctg gaatgagcac cctccaagaa
ANDV Epili     ------taga ctccttgaga agctactgct gcgaaagctg gaatgagcac cctccaagaa
ANDV NRC-2     ---------- ctccttgaga agctactgct gcgaaagctg gaatgagcac cctccaagaa
ANDV NRC-4     --------ga ctccttgaga agctactgct gcgaaagctg gaatgagcac cctccaagaa
ANDV NRC-6     tagtagtaga ctccttgaga agctactgct gcgaaagctg gaatgagcac cctccaagaa

               ....|....| ....|....| ....|....| ....|....| ....|....| ....|....|
                   65         75         85         95        105        115
SNV NM H10     gtgcaagaca acatcactct ccacgaacaa caactcgtga ctgccaggca gaagctcaaa
RIOMV HTN-     gtacaggaag gtatcactct tcatgaacaa cagcttgttg cagcccgcca gaagcttaag
LANV 510B      gtacaggaag gtatcactct tcatgaacaa cagcttgttg cagcccgcca gaagcttaag
CHOV 588       gtgcaggaaa atatcaccct acatgaacag cagcttgtta ctgcacggca aaagcttaaa
JABV AKM96     gtgcaagaaa acatcacact acacgagcag cagctcgtga ctgctcgtca aaagttaaaa
MAPV HV-97     atacaggaaa acatcacagt acacgaacag caattagttg ctgctcggca aaaacttaaa
JUQV oln19     attcaagata gtatcacagt acacgaacaa cagctcgtga ctgccaggca aaaacttaaa
JUQV LH        atccaagaca gcatcacagt acacgaacaa cagctcgtga ctgccaggca gaagcttaaa
PERV 14403     ttgcaggaaa atatcaccgc acacgaacaa catctcgtca ctgccaggca gaaacttaaa
MACV 13796     ttgcaagaga atatcactgc acacgaacaa caactcgtga ctgccaggca gaagctgaaa
ORNV O1229     ctacaggaaa atatcacagc acacgaacaa cagctcgtga ctgctcggca aaagcttaaa
ORNV AND N     ctacaggaaa atatcacagc acacgaacaa cagctcgtga ctgctcggca aaagcttaaa
LECV BMJ-N     ttacaagaaa acatcacagc acacgaacag cagctcgtga ctgctcggca aaagcttaaa
LECV BMJ-O     ttacaagaaa atatcacagc acacgaacaa cagctcgtga ctgctcggca aaagctcaaa
LECV Plata     ttacaagaaa atatcacagc acacgaacaa cagctcgtga ctgctcggca aaagcttaaa
LECV CO22-     ttacaagaaa gtatcacagc acacgaacaa cagctcgtga ctgctcggca aaagcttaaa
LECV BA18-     ttacaagaaa atatcacagc acacgaacaa cagctcgtga ctgctcggca aaagcttaaa
LECV BA18-     ttacaagaaa atatcacagc acacgaacaa cagctcgtga ctgctcggca aaagcttaaa
LECV ER19-     ttacaagaaa atatcacagc acacgaacaa cagctcgtga ctgctcggca aaagcttaaa
LECV 22819     ttacaagaaa atatcacagc acacgaacaa cagctcgtga ctgctcggca aaagcttaaa
BAV BA10-A     ttacaagaaa atatcacagc acacgaacag cagctcgtga ctgctcggca aaagcttaaa
```

```
BAV BA16-L   ttacaagaaa atatcacagc acacgaacaa cagctcgtga ctgctcggca aaagcttaaa
BAV BA02-C   ttacaagaaa atatcacagc acacgaacaa cagctcgtga ctgctcggca aaagcttaaa
BAV BA17-S   ttacaagaaa atatcacagc acacgaacaa cagctcgtga ctgctcggca aaagctcaaa
BAV BA11-A   ttacaagaaa atatcacagc acacgaacaa cagctcgtga ctgctcggca aaagctcaaa
BAV BA02-C   ttacaagaaa atatcacagc acacgaacaa cagctcgtga ctgctcggca aaagcttaaa
BAV BA02-C   ttacaagaaa atatcacagc acacgaacaa cagctcgtga ctgctcggca aaagcttaaa
BAV BA02-C   ttacaagaaa atatcacagc acacgaacaa cagctcgtga ctgctcggca aaagcttaaa
BAV Hu3969   ttacaagaaa atatcacagc acacgaacaa cagctcgtga ctgctcggca aaagcttaaa
ANDV CHI-9   ttgcaggaaa acatcacagc acacgaacaa cagctcgtga ctgctcggca aaagcttaag
ANDV AREB1   ttacaagaaa acatcacagc acacgaacaa cagctcgtga ctgctcggca aaagcttaaa
ANDV Epuye   ctacaagaaa acatcacagc acacgaacaa cagctcgtga ctgctcggca aaagcttaaa
ANDV Epili   ttacaagaaa acatcacagc acacgaacaa cagctcgtga ctgctcggca aaagcttaaa
ANDV NRC-2   ttacaagaaa acatcacagc acacgaacaa cagctcgtga ctgctcggca aaagcttaaa
ANDV NRC-4   ttacaagaaa acatcacagc acacgaacaa cagctcgtga ctgctcggca aaagcttaaa
ANDV NRC-6   ttacaagaaa acatcacagc acacgaacaa cagctcgtga ctgctcggca aaagcttaaa

             ....|....| ....|....| ....|....| ....|....| ....|....| ....|....|
                  125        135        145        155        165        175
SNV NM H10   gatgcagaaa gagcggtgga attggacccc gatgatgtta acaaaagcac attacagagc
RIOMV HTN-   gatgccgaga aagcagttga agtggaccca gatgacgtta acaagagcac actacaaagc
LANV 510B    gatgccgaga aagcagttga agtggaccca gatgacgtta acaagagcac actacaaagc
CHOV 588     gatgccgaga gagcaattga agtggacccg gatgaagtta acaaaagtac actacagggg
JABV AKM96   gatgcagaaa aggcggtgga tgcagaccca gatgatgtca ataaaagtac acttcaaagt
MAPV HV-97   gatgccgaga aggcagtgga ggtggaccca gatgacgtta ataagagcac attacaaaac
JUQV oln19   gatgccgaga aagcagtgga ggtggaccca gatgacgtta acaagagcac attacaaagc
JUQV LH      gatgccgaga aagcagtgga ggtggaccca gatgacgtta acaagagcac attacaaagc
PERV 14403   gatgccgaga agaccgtgga agtggaccca gatgaagtca acaagagcac attacaaagt
MACV 13796   gatgcagaaa aagccgtgga cgtggaccca gatgaggtta acaagagtac actacaaggt
ORNV Ol229   gatgccgaaa agacggtgga ggtggacccg gatgaggtta acaagagcac attacaaagt
ORNV AND N   gatgccgaaa agacggtgga ggtggacccg gatgaggtta acaagagcac attacaaagt
LECV BMJ-N   gatgccgaaa agacggtgga ggtggacccg gatgaggtta acaagagcac actacaaagt
LECV BMJ-O   gatgccgaaa agacggtgga ggtggacccg gatgaggtta acaagagcac actacaaagt
LECV Plata   gatgccgaga agacggtgga ggtggacccg gatgaggtta acaagagcac actacaaagt
LECV CO22-   gatgccgaaa agacggtgga ggtggacccg gatgaggtta acaagagcac actacaaagt
LECV BA18-   gatgccgaaa agacggtgga ggtggacccg gatgaggtta acaagagcac actacaaagt
LECV BA18-   gatgccgaaa agacggtgga ggtggacccg gatgaggtta acaagagcac actacaaagt
LECV ER19-   gatgccgaaa agacggtgga ggtggacccg gatgaggtta acaagagcac actacaaagt
LECV 22819   gatgccgaaa agacggtgga ggtggacccg gatgaggtta acaagagcac actacaaagt
BAV BA10-A   gatgccgaaa agacggtgga ggtggacccg gatgaggtta acaagagcac actacaaagt
BAV BA16-L   gatgccgaaa agacggtgga ggtggacccg gatgaggtta acaagagcac actacaaagt
BAV BA02-C   gatgccgaaa agacggtgga ggtggacccg gatgaggtta acaagagcac actacaaagt
BAV BA17-S   gatgccgaaa agacggtgga ggtggacccg gatgaggtta acaagagcac actacaaagt
BAV BA11-A   gatgccgaaa agacggtgga ggtggacccg gatgaggtta acaagagcac actacaaagt
BAV BA02-C   gatgccgaaa agacggtgga ggtggacccg gatgaggtta acaagagcac actacaaagt
BAV BA02-C   gatgccgaaa agacggtgga ggtggacccg gatgaggtta acaagagcac actacaaagt
BAV BA02-C   gatgccgaaa agacggtgga ggtggacccg gatgaggtta acaagagcac actacaaagt
BAV Hu3969   gatgccgaaa agacggtgga ggtggacccg gatgaggtta acaagagcac actacaaagt
ANDV CHI-9   gatgccgaga aggcagtgga ggtggacccg gatgacgtta acaagagcac actacaaaat
ANDV AREB1   gatgccgaga aggcggtgga ggtggacccg gatgacgtta acaagagcac attacaaagt
ANDV Epuye   gatgccgaga aggcggtgga ggtggacccg gatgacgtta acaagagcac attacaaagt
ANDV Epili   gatgccgaga aggcggtgga ggtggacccg gatgacgtta acaagagcac actacaaagt
ANDV NRC-2   gatgccgaga aggcggtgga ggtggacccg gatgacgtta acaagagcac actacaaagt
ANDV NRC-4   gatgccgaga aggcggtgga ggtggacccg gatgacgtta acaagagcac actacaaagt
ANDV NRC-6   gatgccgaga aggcggtgga ggtggacccg gatgacgtta acaagagcac attacaaagt

             ....|....| ....|....| ....|....| ....|....| ....|....| ....|....|
                  185        195        205        215        225        235
SNV NM H10   agacgggcag ctgtgtctgc attggagacc aaactcggag aacttaagcg ggaactggct
RIOMV HTN-   agacgggcag ctgtgtctgc attggagaat aaactcgcag aactcaagag gcagttggca
LANV 510B    agacgggcag ctgtgtctgc attggagaat aaactcgcag aactcaagag gcagttggca
CHOV 588     agaagggcag ctgtgtctac actggaaacc aagctcggag aactcaaaag gcagctggca
JABV AKM96   agaaaggcag cagtttctgc tcttgagaca aaattagggg agctgaaaag acaactggct
MAPV HV-97   agacgggcag ctgtgtctac attggagagt aaactcgggg aactcaaaag gcaactggca
JUQV oln19   agacgggcag ctgtgtctac attggagact aaactcgggg aactcaagcg gcaactggca
```

```
JUQV LH       agacgggcag ctgtgtctac attggagact aaactcgggg aactcaagcg acaactggca
PERV 14403    agacgggcgg ctgtgtctac attggagacc aaacttggag agctcaagag gcagcttgca
MACV 13796    cgacgggcag ctgtgtcaac attggagacc aaacttggag agctcaagag acagcttgct
ORNV 01229    agacgggcag ctgtgtctac attggagacc aaactcggag aacttaagag gcagcttgcg
ORNV AND N    agacgggcag ctgtgtctac attggagacc aaactcggag aacttaagag gcagcttgcg
LECV BMJ-N    agacgggcag ctgtgtctac attggagacc aaactcgggg aactcaagag acaacttgca
LECV BMJ-O    agacgggcag ctgtgtctac attggagacc aagctcgggg aactcaagag acaacttgca
LECV Plata    agacgggcag ctgtgtctac attggagacc aaactcgggg aacttaagag acaacttgca
LECV CO22-    agacgggcag ctgtgtctac actggagacc aaacttgggg aactcaagag acaacttgca
LECV BA18-    agacgggcag ctgtgtctac actggagacc aaacttgggg aactcaagag acaacttgcc
LECV BA18-    agacgggcag ctgtgtctac actggagacc aaactcgggg aactcaagag acaacttgcc
LECV ER19-    agacgggcag ctgtgtctac actggagacc aaactcgggg aactcaagag acaacttgca
LECV 22819    agacgggcag ctgtgtctac actggagacc aaactcgggg aactcaagag acaacttgca
BAV BA10-A    agacgggcag ctgtgtctac attggagacc aaactcggag aactcaagag gcagcttgcc
BAV BA16-L    agacgggcag ctgtgtctac attggagacc aaactcggag aactcaagag gcagcttgcc
BAV BA02-C    agacgggcag ctgtgtctac attggagacc aaactcggag aactcaagag gcagcttgcc
BAV BA17-S    agacgggcag ctgtgtctac attggagacc aaactcggag aactcaagag gcagcttgcc
BAV BA11-A    agacgggcag ctgtgtctac attggagacc aaactcggag aactcaagag gcagcttgcc
BAV BA02-C    agacgggcag ctgtgtctac attggagacc aaactcggag aactcaagag gcagcttgcc
BAV BA02-C    agacgggcag ctgtgtctac attggagacc aaactcggag aactcaagag gcagcttgcc
BAV BA02-C    agacgggcag ctgtgtctac attggagacc aaactcggag aactcaagag gcagcttgcc
BAV Hu3969    cgacgggcag ctgtgtctac attggagacc aaactcggag aactcaagag gcagcttgcc
ANDV CHI-9    agacgggcag ctgtgtctac attggagacc aaactcggag aacttaagag gcaacttgca
ANDV AREB1    agacgggcag ctgtgtctac attggagacc aaactcgggg aactcaagag gcaacttgcg
ANDV Epuye    agacgggcag ctgtgtctac attggagacc aaactcgggg aactcaagag gcaacttgcg
ANDV Epili    agacgggcag ctgtgtctac attggagacc aaactcgggg aactcaagag gcaacttgcg
ANDV NRC-2    agacgggcag ctgtgtctac attggagacc aaactcgggg aactcaagag gcaacttgca
ANDV NRC-4    agacgggcag ctgtgtctac actggagacc aaactcgggg aactcaagag gcaacttgca
ANDV NRC-6    agacgggcag ctgtgtctac attggagacc aaactcgggg aactcaagag gcaacttgcg

              ....|....| ....|....| ....|....| ....|....| ....|....| ....|....|
                  245        255        265        275        285        295
SNV NM H10    gatcttattg cagctcagaa attggcttca aaacctgttg atccaacagg gattgaacct
RIOMV HTN-    gatttggttg cagctcaaaa attggctgca aaaccagttg atccaacagg gcttgaacct
LANV 510B     gatttggttg cagctcaaaa attggctgca aaaccagttg atccaacagg gcttgaacct
CHOV 588      gatttggtgg cagctcaaaa attggcttca aaaccagttg atccaacagg gattgagcct
JABV AKM96    gatctggtag catctcaaaa attagcctcc aaaccagttg accccacagg gttagaacct
MAPV HV-97    gatcttgtgg cagctcagaa actggcttca aaaccagttg atccaacagg gcttgaacct
JUQV oln19    gatttggttg cagctcagaa actggctaca aaaccagttg atccaacagg gcttgagcct
JUQV LH       gatctggttg cagctcagaa actggctaca aaaccagttg atccaacagg gcttgagcct
PERV 14403    gatttggtgg cagctcaaaa gctggctgca aagccagttg atccaacagg gcttgagcct
MACV 13796    gacttggtgg cagctcaaaa actggctaca aaaccagttg atccaacagg gattgagcct
ORNV 01229    gatctggtgg cagctcaaaa actggctaca aaaccagttg atccaacagg gcttgagcct
ORNV AND N    gatctggtgg cagctcaaaa actggctaca aaaccagttg atccaacagg gcttgagcct
LECV BMJ-N    gatctggtgg cagctcaaaa actggctaca agaccagttg atccaacagg gcttgagcct
LECV BMJ-O    gatttggtgg cagctcaaaa actggctaca aaaccagttg atccaacagg gcttgagcct
LECV Plata    gatttggtgg cagctcaaaa actggctaca aaaccagttg atccaacagg gcttgagcct
LECV CO22-    gatctggtgg cagctcaaaa actggctaca aaaccagttg atccaacagg gcttgagcct
LECV BA18-    gatctggtgg cagctcaaaa actggctaca aaaccagttg atccaacagg gcttgagcct
LECV BA18-    gatctggtgg cagctcaaaa actggctaca aaaccagttg atccaacagg gcttgagcct
LECV ER19-    gatctggtgg cagctcaaaa actggctaca aaaccagttg atccaacagg gcttgagcct
LECV 22819    gatctggtgg cagctcaaaa actggctaca aaaccagttg atccaacagg gcttgagcct
BAV BA10-A    gatctggtgg cagctcaaaa actggctaca aaaccagttg atccaacagg gcttgagcct
BAV BA16-L    gatttggtgg cagctcaaaa actggctaca aaaccagttg atccaacagg gcttgagcct
BAV BA02-C    gatttggtgg cagctcaaaa actggctaca aaaccagttg atccaacagg gcttgagcct
BAV BA17-S    gatttggtgg cagctcaaaa actggctaca aaaccagttg atccaacagg gcttgagcct
BAV BA11-A    gatttggtgg cagctcaaaa actggctaca aaaccagttg atccaacagg gcttgagcct
BAV BA02-C    gatttggtgg cagctcaaaa actggctaca aaaccagttg atccaacagg gcttgagcct
BAV BA02-C    gatttggtgg cagctcaaaa actggctaca aaaccagttg atccaacagg gcttgagcct
BAV BA02-C    gatttggtgg cagctcaaaa actggctaca aaaccagttg atccaacagg gcttgagcct
BAV Hu3969    gatttggtgg cagctcaaaa actggctaca aaaccagttg atccaacagg gcttgagcct
ANDV CHI-9    gatttggtgg cagctcaaaa attggctaca aaaccagttg atccaacagg gcttgagcct
ANDV AREB1    gatctggtgg cagctcaaaa gttggctaca aaaccagttg atccaacagg gcttgagcct
ANDV Epuye    gatctggtgg cagctcaaaa gttggctaca aaaccagttg atccaacagg gcttgagcct
```

```
ANDV Epili    gatctggtgg cagctcaaaa gttggctaca aaaccagttg atccaacagg gcttgagcct
ANDV NRC-2    gatctggtgg cagctcaaaa attggctaca aaaccagttg atccaacagg gcttgagcct
ANDV NRC-4    gatctggtgg cagctcaaaa attggctaca aaaccagttg atccaacagg gcttgagcct
ANDV NRC-6    gatctggtgg cagctcaaaa gttggctaca aaaccagttg atccaacagg gcttgagcct

              ....|....| ....|....| ....|....| ....|....| ....|....| ....|....|
                  305        315        325        335        345        355
SNV NM H10    gatgaccatc taaaggaaaa gtcatcattg agatatggaa atgtccttga tgtaaattcc
RIOMV HTN-    gatgaccatc tgaaggaaaa gtctgctctg cgttatggaa atgtcctgga tgtcaatgct
LANV 510B     gatgaccatc tgaaggaaaa gtctgctctg cgttatggaa atgtcctgga tgtcaatgct
CHOV 588      gatgaccatt tgaaggaaaa atctacactt cgttatggca atgtcttgga tgtgaactcc
JABV AKM96    gatgaccatc tgaaggaaaa atcaacactg cgatatggaa atgtcattga tgtcaactcc
MAPV HV-97    gatgaccatt tgaaggaaaa atcatcactg cgttatggaa atgtccttga tgtcaactcg
JUQV oln19    gatgaccatc taaaggagaa gtcttcatta cggtatggca atgtcatcga tgtcaaccca
JUQV LH       gatgaccatc tgaaggagaa gtcttcctta cggtatggca atgtcatcga tgtcaaccca
PERV 14403    gatgaccatc tgaaggagaa atcatctttg aggtatggga atgtcttgga tgtcaactcc
MACV 13796    gatgaccatc tgaaggaaaa atcatccttg agatatggga atgtccttga tgtcaattct
ORNV Ol229    gatgatcatc tgaaagaaaa atcatctctc cgttatggga atgtcctgga tgtcaactcc
ORNV AND N    gatgatcatc taaaagagaa atcatctctc cgttatggga atgtcctgga tgtcaactcc
LECV BMJ-N    gatgatcatc taaaggaaaa gtcatcctta aggtatggca acgtcctgga tgtcaactcc
LECV BMJ-O    gatgatcatc taaaggaaaa gtcatcctta aggtatggca atgtcctgga tgtcaactcc
LECV Plata    gatgatcatc taaaggaaaa gtcatccttg aggtatggca atgttctgga tgtcaactcc
LECV CO22-    gatgatcatc taaaggaaaa gtcatccctg agatatggca atgttctgga tgtcaactcc
LECV BA18-    gatgaccatc taaaggagaa gtcatccctg agatatggta atgttctgga tgtcaactct
LECV BA18-    gatgaccatc taaaggaaaa gtcatccctg agatatggca atgttctgga tgtcaactct
LECV ER19-    gatgatcatc taaaggaaaa gtcatccctg agatatggca atgttctgga tgtcaactct
LECV 22819    gatgatcatc taaaggaaaa gtcatccctg agatatggta atgttctgga tgtcaactct
BAV BA10-A    gatgatcatt tgaaggaaaa gtcatcattg cgttatggga atgtgctgga tgtcaactcc
BAV BA16-L    gatgatcatt tgaaggaaaa atcatcattg cgttatggga atgtactgga tgtcaactcc
BAV BA02-C    gatgatcatt tgaaggaaaa atcatcattg cgttatggaa atgtgctgga tgtcaactcc
BAV BA17-S    gatgatcatt tgaaggaaaa atcatcattg cgttatggga atgtgctgga tgtcaactcc
BAV BA11-A    gatgatcatt tgaaggaaaa atcatcattg cgttatggga atgtgctgga tgtcaactcc
BAV BA02-C    gatgatcatt tgaaggaaaa atcatcattg cgttatggga atgtgctgga tgtcaactcc
BAV BA02-C    gatgatcatt tgaaggaaaa atcatcattg cgttatggga atgtgctgga tgtcaactcc
BAV BA02-C    gatgatcatt tgaaggaaaa atcatcattg cgttatggga atgtgctgga tgtcaactcc
BAV Hu3969    gatgatcatt tgaaggaaaa gtcatcattg cgttacggga atgtgctgga tgtcaactcc
ANDV CHI-9    gatgatcatc taaaggaaaa atcatctctg agatatggga atgtcctgga tgttaattca
ANDV AREB1    gatgaccatc tgaaagagaa atcatctctg agatatggga atgtcctgga tgttaactca
ANDV Epuye    gatgaccatc tgaaagagaa atcatctctg agatatggga atgtcctgga tgttaactca
ANDV Epili    gatgaccatc tgaaagaaaa atcatctctg agatatggga atgtcctgga tgttaactca
ANDV NRC-2    gatgaccatc tgaaagagaa atcatccctt agatatggga atgtcctgga tgttaactca
ANDV NRC-4    gatgaccatc tgaaagagaa atcatctctg agatatggga atgtcctgga tgtgaattca
ANDV NRC-6    gatgaccatc tgaaagagaa atcatctcta agatatggga atgtcctgga tgttaactca

              ....|....| ....|....| ....|....| ....|....| ....|....| ....|....|
                  365        375        385        395        405        415
SNV NM H10    attgacttag aagagccaag tgggcaaaca gctgattgga aatccatcgg actctacatt
RIOMV HTN-    attgaccttg aagaaccgag tggccaaact gccgattgga aggctattgg agcctatatt
LANV 510B     attgaccttg aagaaccgag tggccaaact gccgattgga aggctattgg agcctatatt
CHOV 588      attgatcttg aagagcctag tggtcaaaca gctgattgga ggtcaattgg tgcttatatc
JABV AKM96    attgacttgg aagaacctag tggccagaca gcagattgga gggtcattgg tgcctacatc
MAPV HV-97    attgatctgg aagagccaag tgggcaaaca gcagattggt tgtcaattgg gatgtacatc
JUQV oln19    attgatctgg aagagcccag tggccaaact gccgattgga aagcaattgg tgcctatatc
JUQV LH       attgatctgg aagaacccag tggccagact gcagactgga aagcaattgg tgcctatatc
PERV 14403    attgaccttg aagaaccgag tggtcaaaca gctgattgga aagcaattgg ggcttatata
MACV 13796    attgacctgg aagaaccaag tggacaaacc gctgactgga aggctattgg agcatacata
ORNV Ol229    attgatcttg aagagccaag tggtcaaact gctgattgga aagctattgg ggcctacata
ORNV AND N    attgatcttg aagagccaag tggtcaaact gctgattgga aagctattgg ggcctacata
LECV BMJ-N    attgatctgg aagaaccaag tgggcagact gccgactgga ggtctattgg agcttatata
LECV BMJ-O    attgatctgg aagaaccaag tggacagact gctgattgga ggtctattgg ggcttatata
LECV Plata    attgatcttg aagaaccgag tggacaaact gccgattgga ggtctatcgg ggcttacata
LECV CO22-    attgatctag aagaaccaag tggacagact gctgattgga gatctatcgg ggcttatata
LECV BA18-    atcgatctag aagaaccaag tggacagact gctgattgga gatctatcgg ggcatatata
LECV BA18-    attgatctag aagaaccaag tggacagact gctgattgga gatctatcgg ggcatatata
```

```
LECV ER19-   attgatctag aagaaccaag tggacagact gctgattgga gatctatcgg ggcatatata
LECV 22819   attgatctag aagaaccaag tggacagact gctgattgga gatctattgg ggcatatata
BAV BA10-A   attgatttgg aagaaccaag tggccaaact gctgattgga gggctattgg agcttacata
BAV BA16-L   attgatctgg aagaaccgag tggccaaact gctgattgga gggctattgg agcttacata
BAV BA02-C   attgatctgg aagaaccgag tggccaaact gctgattgga gggctattgg agcttacata
BAV BA17-S   attgatctgg aagaaccgag tggccaaact gctgattgga gggctattgg agcttacata
BAV BA11-A   attgatctgg aggaaccgag tggccagact gctgattgga gggctattgg agcttacata
BAV BA02-C   attgatctgg aagaaccgag tggccaaact gctgattgga gggctattgg agcttacata
BAV BA02-C   attgatctgg aagaaccgag tggccaaact gctgattgga gggctattgg agcttacata
BAV BA02-C   attgatctgg aagaaccgag tggccaaact gctgattgga gggctattgg agcttacata
BAV Hu3969   attgatctgg aagaaccaag tggccaaact gctgactgga gggctattgg agcttacata
ANDV CHI-9   attgatttgg aagaaccgag tggacagact gctgattgga aggctatagg agcatacatc
ANDV AREB1   attgacttgg aagaaccgag tggacagact gctgattgga aggctatagg agcatatatc
ANDV Epuye   atcgacttgg aagaaccgag tggacagact gctgattgga aggctatagg agcatatatc
ANDV Epili   attgacttgg aagaaccgag tggacagact gctgattgga aggctatagg agcatatatt
ANDV NRC-2   atcgatttgg aagaaccgag tggacagact gctgattgga aggctatagg agcatatatc
ANDV NRC-4   attgatttgg aagaaccaag tggacagact gctgattgga aggctatagg agcatatatc
ANDV NRC-6   attgacttgg aagaaccgag tggacagact gctgattgga aggctatagg agcatatatc

             ....|....| ....|....| ....|....| ....|....| ....|....| ....|....|
                  425        435        445        455        465        475
SNV NM H10   ctaagtttcg cattaccgat tattcttaaa gccttgtaca tgttatctac taggggccgt
RIOMV HTN-   ctaggatttg taataccaat tatcctaaag gcattatata tgctttcaac aagagggagg
LANV 510B    ctaggatttg taataccaat tatcctaaag gcattatata tgctttcaac aagagggagg
CHOV 588     ctcggatttg cactaccaat catcttgaaa gccctctaca tgttatccac aagagggagg
JABV AKM96   ttgagctttg caattccaat catacttaaa gctctgtaca tgctctccac aagaggaagg
MAPV HV-97   ttgggctttg ctatccccat catattgaag gcactttaca tgttatccac aagagggaga
JUQV oln19   ttgggttttg caataccgat aatacttaaa gcattgtata tgctttcaac ccgagggaga
JUQV LH      ttaggttttg caataccaat aatacttaaa gcactgtaca tgctttcaac ccgagggaga
PERV 14403   ctgggatttg caattccaat catactgaaa gcactataca tgttatccac ccgtggaagg
MACV 13796   ctgggttttg caattccgat catcctaaag gctttgtaca tgctgtcaac ccgtggaagg
ORNV O1229   ttaggatttg ccataccaat catcctgaaa gctctctaca tgctttccac ccgtgggaga
ORNV AND N   ttaggatttg ccataccaat catcctgaaa gctctctaca tgctttccac ccgtgggaga
LECV BMJ-N   ctaggctttg caatcccaat tatcctaaaa gccttatata tgctttctac cagagggcgg
LECV BMJ-O   ctaggctttg caatcccaat catcctaaag gccttgtata tgctttctac ccgcgggcgt
LECV Plata   ctagggtttg caataccaat tatcctaaag gccttgtata tgctctccac ccggggtgg
LECV CO22-   cttggatttg caataccgat tatcctcaag gccttgtata tgctttctac ccgggggcgg
LECV BA18-   cttggatttg caataccaat tatcctcaag gccctgtata tgctttctac ccgggggcgg
LECV BA18-   cttgggtttg caataccaat tatcctcaag gctttgtata tgctttctac ccgggggagg
LECV ER19-   cttgggtttg caataccaat tatccttaag gccctgtata tgctctctac ccgggggcgg
LECV 22819   cttgggtttg caataccaat tatcctcaag gccctatata tgctttctac ccgggggcgg
BAV BA10-A   cttggatttg ctattcccat catccttaaa gctctatata tgctttccac tcgtgggcga
BAV BA16-L   cttggatttg ctattcctat tatccttaaa gctctatata tgctttctac tcgtgggcga
BAV BA02-C   cttggatttg ctattcctat catccttaaa gctctatata tgctttctac tcgtgggcga
BAV BA17-S   cttggatttg ctattcctat catccttaaa gctctatata tgctttctac tcgtgggcga
BAV BA11-A   cttggatttg ctattcctat catccttaaa gctctatata tgctttctac tcgtgggcga
BAV BA02-C   cttggatttg ctattcctat catccttaaa gctctatata tgctttctac tcgtgggcga
BAV BA02-C   cttggatttg ctattcctat catccttaaa gctctatata tgctttctac tcgtgggcga
BAV BA02-C   cttggatttg ctattcctat catccttaaa gctctatata tgctttctac tcgtgggcga
BAV Hu3969   cttggatttg ctattcctat catccttaaa gctctatata tgctttctac tcgtgggcga
ANDV CHI-9   ttagggtttg caattccgat catcctaaag gccttataca tgctgtcaac ccgtgggaga
ANDV AREB1   ttagggtttg caattccgat catactaaaa gccctataca tgctgtcaac ccgcgggaga
ANDV Epuye   ttagggtttg caattccgat catactaaaa gccctatata tgctgtcaac ccgcgggaga
ANDV Epili   ttagggtttg caattccgat catactaaaa gccctatata tgctgtcaac ccgcgggaga
ANDV NRC-2   ttagggtttg caattccgat catactaaaa gccctatata tgctgtcaac ccgtggaaga
ANDV NRC-4   ttagggtttg caattccgat catactaaaa gccctatata tgctatcaac ccgtgggaga
ANDV NRC-6   ttagggtttg caattccgat catactaaaa gccctatata tgctgtcaac ccgcgggaga

             ....|....| ....|....| ....|....| ....|....| ....|....| ....|....|
                  485        495        505        515        525        535
SNV NM H10   caaacaatca aagaaaacaa gggaacaaga attcgattca aggatgattc atcttatgaa
RIOMV HTN-   cagactgtta aagagaacaa agggaccagg attcgattca aggatgattc atcatttgaa
LANV 510B    cagactgtta aagagaacaa agggaccagg attcgattca aggatgattc atcatttgaa
CHOV 588     cagactgtga aagaaaacaa aggcaccagg atcagattta aagatgattc atccttcgaa
```

```
JABV AKM96   cagacagtta aagagaataa agggacaagg atcaggttca aggatgattc ctcttatgaa
MAPV HV-97   cagacagtaa aggaaaacaa agggacccgg atcaggttca aggatgattc ctcttttgaa
JUQV oln19   caaacagtga aggacaacaa atctactagg attaggttca aagatgattc atcattcgag
JUQV LH      cagacagtaa aggacaataa agggactagg atcaggttta aagatgactc atccttcgaa
PERV 14403   caaacagtta aggacaacaa aggcaccaga ataaggttca aagatgattc atcctttgaa
MACV 13796   cagacggtca aggacaacaa ggggactaga attagattca aggatgattc atcttttgaa
ORNV O1229   caaactgtca aagacaacaa ggggactaga ataaggttca aggatgactc atcatttgaa
ORNV AND N   cagactgtca aagacaacaa ggggactaga ataaggttca aggatgactc atcatttgaa
LECV BMJ-N   cagactgtca aggacaacaa agggaccagg atacggttca aggatgattc atcttttgaa
LECV BMJ-O   cagactgtca aagacaataa agggactaga atacggttta aggatgattc atcatttgaa
LECV Plata   cagactgtta aggataacaa agggacaagg atacggttca aggacgactc atcatttgag
LECV CO22-   cagactgtca aggacaacaa agggacaaga atacggttta aagatgattc atcatttgag
LECV BA18-   cagactgtca aggacaacaa agggacaaga atccggttta aagatgattc atcttttgag
LECV BA18-   cagactgtca aggacaacaa agggacaaga atccggttta aagatgattc atctttcgag
LECV ER19-   cagactgtca aggacaacaa agggacaaga atccggttta aagatgattc atcatttgag
LECV 22819   cagactgtca aggacaacaa agggacaaga atccggttta aagatgattc ttcatttgag
BAV BA10-A   cagactgtca aagacaacaa aggaaccaga ataaggttca aggatgattc atcatttgag
BAV BA16-L   cagactgtca aagacaacaa aggaaccagg ataagattta aggatgactc atcatttgag
BAV BA02-C   cagactgtta aagacaacaa aggaaccagg ataagattta aggatgactc atcatttgag
BAV BA17-S   cagactgtta aagacaacaa aggaaccagg ataagattta aggatgactc atcatttgag
BAV BA11-A   cagactgtta aagacaacaa aggaaccagg ataagattta aggatgactc atcatttgag
BAV BA02-C   cagactgtca aagacaacaa aggaaccaga ataagattta aggatgactc atcatttgag
BAV BA02-C   cagactgtca aagacaacaa aggaaccaga ataagattta aggatgactc atcatttgag
BAV BA02-C   cagactgtca aagacaacaa aggaaccagg ataagattta aggatgactc atcatttgag
BAV Hu3969   cagactgtca aagacaacaa aggaaccagg ataagattca aagatgactc atcatttgag
ANDV CHI-9   caaactgtga aagacaacaa agggaccagg ataaggttta aggatgattc ttcctttgaa
ANDV AREB1   caaactgtga aagacaataa aggaaccagg ataagattta aggatgattc ttcctttgaa
ANDV Epuye   caaactgtga aagacaataa aggaactagg ataaggttta aggatgattc ttcctttgaa
ANDV Epili   caaactgtga aagacaataa aggaactagg ataaggttta aggatgattc ttcctttgaa
ANDV NRC-2   cagactgtga aggacaacaa aggaaccagg ataaggttta aggatgattc ttcctttgaa
ANDV NRC-4   cagactgtga aagacaacaa aggaaccagg ataaggttta aggatgattc ttcctttgaa
ANDV NRC-6   caaactgtga aagacaataa aggaactagg ataaggttta aggatgattc ttcctttgaa

             ....|....| ....|....| ....|....| ....|....| ....|....| ....|....|
                  545        555        565        575        585        595
SNV NM H10   gaagtcaatg ggatacgtaa gccaagacat ctgtatgttt ctatgccaac tgcccagtct
RIOMV HTN-   gaagtcaatg gcatccgaaa acctaaacac ttgtatgtgt caatgcctac tgcacaatct
LANV 510B    gaagtcaatg gcatccgaaa acctaaacac ttgtatgtgt caatgcctac tgcacaatct
CHOV 588     gaggtaaatg ggataagaaa gcctaaacat ctttatgtgt cgatgcccac tgcacagtca
JABV AKM96   gaaatcaatg ggataaggaa accaaagcat ctgtatgtat ccatgccaac tgcacaatca
MAPV HV-97   gaagttaatg gcatcaggaa accaaaacat ctttatgtct ctatgccaac agcacagtca
JUQV oln19   gaagtcaatg ggatccggaa accaaaacat ctttatgttt caatgccaac tgcacaatcg
JUQV LH      gaggtcaatg gaatccggaa accaaagcat ctttatgttt caatgccaac tgcacaatca
PERV 14403   gaggtaaatg ggatccggaa gccgaaacat ctttatgttt caatgccaac agcacagtcc
MACV 13796   gaagtaaatg ggattaggaa gccaaagcat ctctacatct ccatgccgac tgctcagtcc
ORNV O1229   gaggtcaacg gtatccggaa accgaagcat ctctatgtgt caatgccaac tgcacaatca
ORNV AND N   gaggtcaacg gtatccggaa accgaagcat ctctatgtgt caatgccaac tgcacaatca
LECV BMJ-N   gaggtcaatg gcatccgaaa gcccaagcat ttgtatgtat ccatgccaac agcacaatca
LECV BMJ-O   gaggtcaatg gcatccgaaa gcccaagcac ttgtatgtgt ccatgccaac agcacagtca
LECV Plata   gaggttaatg gcatccgaaa gcctaagcat ctgtatgtgt ctatgcccac agcacagtca
LECV CO22-   gaggtcaatg gcatccgaaa gcctaagcat ttgtatgtat ccatgcccac agcacaatca
LECV BA18-   gaggtcaatg gcatccgaaa gcctaaacat ttgtatgtat ccatgcccac agcacaatca
LECV BA18-   gaggtcaacg gcatccgaaa gcctaaacat ctgtatgtat ccatgcctac agcacaatca
LECV ER19-   gaggtcaatg gcatccgaaa gcctaaacat ttgtatgtat ccatgcctac agcacaatca
LECV 22819   gaggtcaatg gcatccgaaa gcctaaacat ttgtatgtat ccatgcctac agcacaatca
BAV BA10-A   gaggttaatg gcattaggaa gcccaaacat ttatatgtgt caatgccaac agcacaatca
BAV BA16-L   gaggttaacg gcattaggaa acccaaacat ttatatgtgt caatgccaac agcacaatca
BAV BA02-C   gaggtcaacg gcattaggaa gcccaaacat ttatatgtgt caatgccaac agcacaatca
BAV BA17-S   gaggtcaacg gcattaggaa gcccaaacat ttatatgtgt caatgccaac agcacaatca
BAV BA11-A   gaggtcaacg gcattaggaa gcccaaacat ttatatgtgt caatgccaac agcacaatca
BAV BA02-C   gaggttaacg gcattaggaa gcctaaacat ttatatgtgt caatgccaac agcacaatca
BAV BA02-C   gaggttaacg gcattaggaa gcctaaacat ttatatgtgt caatgccaac agcacaatca
BAV BA02-C   gaggttaacg gcattaggaa gcctaaacat ttatatgtgt caatgccaac agcacaatca
BAV Hu3969   gaggtcaacg gcattaggaa gcccaagcat ttatatgtgt caatgccaac agcacaatca
```

```
ANDV CHI-9   gaagtcaatg ggatacgtaa accaaaacac ctttacgtct caatgccaac tgcacagtcc
ANDV AREB1   gaagttaatg ggatacgtaa accaaaacac ctttatgtct caatgccgac tgcacagtct
ANDV Epuye   gaagtcaatg ggatacgtaa accaaaacac ctttatgtct caatgccgac tgcacagtct
ANDV Epili   gaagtcaatg ggatacgtaa accaaaacac ctttatgtct caatgccgac tgcacagtct
ANDV NRC-2   gaggtcaatg ggatacgtaa accgaaacat ctttatgtct caatgccaac tgcacaatct
ANDV NRC-4   gaggtcaacg ggatacgtaa gccgaaacac ctttatgtct caatgccaac tgcacaatct
ANDV NRC-6   gaagtcaatg ggatacgtaa accaaaacac ctttatgtct caatgccgac tgcacagtct

             ....|....| ....|....| ....|....| ....|....| ....|....| ....|....|
                   605        615        625        635        645        655
SNV NM H10   acaatgaaag cagatgagat tactcccggg aggttccgta caattgcttg tggattattc
RIOMV HTN-   acaatgaagg cagatgagat aacaccgggg aggtttagga caattgcatg tggcttattt
LANV 510B    acaatgaagg cagatgagat aacaccgggg aggtttagga caattgcatg tggcttattt
CHOV 588     acaatgaagg ctgatgaaat aacacctgga aggttccgga caattgcatg tggccttttc
JABV AKM96   actatgaagg cagacgagat aacaccaggg aggttcagaa caattgcttg tggtctgttt
MAPV HV-97   actatgaagg ctgatgagat aacaccaggg agatttcgga cgattgcctg tggtcttttt
JUQV oln19   acaatgaagg ctgaagagat aacacctggg cgctttcgga cgattgcttg cggactcttc
JUQV LH      acaatgaagg ctgaagagat aacacctggg cggtttcgaa caattgcttg tggccttttc
PERV 14403   acaatgaaag ctgaagaaat aacaccaggc aggtttagaa ctattgcttg tgggcttttc
MACV 13796   acaatgaaag ctgaggagat aacacctggg aggtttagaa cgattgtatg tggattgttt
ORNV O1229   acaatgaagg ctgaagagat aacacctgga cggttcagga caattgcatg tggactcttc
ORNV AND N   acaatgaagg ctgaagagat aacacctgga cggttcagga caattgcatg tggactcttc
LECV BMJ-N   acaatgaagg ctgaggagat aacaccaggt agacttagaa caattgcatg tggtctattc
LECV BMJ-O   acaatgaagg ctgaggagat aacaccaggg aggttcagaa caattgcatg tggcctcttc
LECV Plata   acaatgaagg ctgaagaaat aacaccaggc agattcagaa cgattgcatg tggtctcttc
LECV CO22-   acaatgaagg ctgaagaaat aacaccaggc agattcagaa caattgcatg tggtctcttc
LECV BA18-   acaatgaagg ctgaagagat aacaccaggc agattcagaa caattgcatg tggtcttttt
LECV BA18-   acaatgaagg ctgaagagat aacaccaggc agattcagaa caattgcatg tggtcttttt
LECV ER19-   acaatgaagg ctgaagagat aacacctggc agattcagaa caattgcatg tggtcttttt
LECV 22819   acaatgaagg ctgaagagat aacaccaggc agatttagaa caattgcatg tggtcttttt
BAV BA10-A   acaatgaagg cagaggaaat aacaccaggt aggttcagaa caattgcatg tggcctcttc
BAV BA16-L   acaatgaagg cagaggaaat aacaccaggt aggttcagaa caattgcatg tggcctcttc
BAV BA02-C   acaatgaagg cagaggaaat aacaccaggt aggttcagaa cgattgcatg tggcctcttc
BAV BA17-S   acaatgaagg cagaggaaat aacaccaggt aggttcagaa cgattgcatg tggcctcttc
BAV BA11-A   acaatgaagg cagaggaaat aacaccaggt aggttcagaa cgattgcatg tggcctcttc
BAV BA02-C   acaatgaagg cagaggaaat aacaccaggt aggttcagaa cgattgcatg tggcctcttc
BAV BA02-C   acaatgaagg cagaggaaat aacaccaggt aggttcagaa cgattgcatg tggcctcttc
BAV BA02-C   acaatgaagg cagaggaaat aacaccaggt aggttcagaa cgattgcatg tggcctcttc
BAV Hu3969   acaatgaagg cagaggaaat aacaccaggt aggttcagaa cgattgcatg tggcctcttc
ANDV CHI-9   actatgaagg ctgaagaaat cacgccagga cgatttagga caattgcttg tggccttttt
ANDV AREB1   actatgaagg ctgaagaaat cacgccagga cgttttagga caattgcttg tggcctcttc
ANDV Epuye   actatgaagg ctgaagaaat cacgccagga cgttttagga caattgcttg tggcctcttc
ANDV Epili   actatgaagg ctgaagaaat cacaccagga cgttttagga caattgcttg tggcctcttc
ANDV NRC-2   actatgaagg ctgaagaaat cacgccagga cgatttagga caattgcttg tggccttttc
ANDV NRC-4   actatgaagg ctgaagaaat cacaccagga cggtttagga caattgcttg tggcctcttt
ANDV NRC-6   actatgaagg ctgaagaaat cacgccagga cgttttagga caattgcttg cggcctcttc

             ....|....| ....|....| ....|....| ....|....| ....|....| ....|....|
                   665        675        685        695        705        715
SNV NM H10   ccagcccaag tcaaagcaag gaatattatc agtcctgtca tgggtgtgat tggctttagt
RIOMV HTN-   cctgctcaaa tcaaagctcg gaacattata agtccagtca tgggtgtcat tggctttggt
LANV 510B    cctgctcaaa tcaaagctcg gaacattata agtccagtca tgggtgtcat tggctttggt
CHOV 588     ccagctcaag tcaaagccag gaatataatt agcccagtaa tgggtgtcat cggttttggt
JABV AKM96   cctgcacagg taaaggcaag aaatataata agtcctgtca tggggggtaat cggatttggc
MAPV HV-97   cctgcacagg tgaaggcaag gaatataatc agtcctgtga tgggtgtcat tgggtttagc
JUQV oln19   ccagcccaag tgaaagcaag gaatattata agtcctgtca tgggtgtcat tggattcagc
JUQV LH      ccagcccaag tgaaggcaag gaatatcata agtcctgtaa tgggagtcat tggattcagc
PERV 14403   cctgcacaag ttaaggccag gaatataatt agtccagtca tgggggtaat tggtttcagc
MACV 13796   cctgctcaag tgaaagcccg gaatatcata agcccagtga tgggggtcat tggttttagt
ORNV O1229   cctgcacaag tgaaagccag gaacatcatc agtccagtca tgggagtaat cggtttagc
ORNV AND N   cctgcacaag tgaaagccag gaacatcatc agcccagtca tgggagtaat cggtttagc
LECV BMJ-N   cctgcacaag tcaaggcaag gagcattatc agtcctgtca tgggagtcat cggatttagc
LECV BMJ-O   cctgcacaag ttaaggcaag aaacatcatt agtcctgtca tgggagttat tggatttagc
LECV Plata   cctgcacaag tgaaggcaag gaacatcatt agtcctgtca tgggggtcat tgggttcagt
```

```
LECV CO22-   cctgcacaag tcaaagctag gaacatcatt agtcctgtca tgggggtcat tggattcagc
LECV BA18-   cctgcacaag tcaaagctag aaacatcatt agtcctgtca tgggggtcat tggattcagc
LECV BA18-   cctgcacaag tcaaagctag aaacatcatt agtcctgtca tgggggtcat tggattcagc
LECV ER19-   cctgcacaag ttaaagctag aaacatcatt agtcctgtca tgggggtcat tggattcagc
LECV 22819   cctgcacaag tcaaagctag aaacatcatt agtcctgtca tgggggtcat tggattcagc
BAV BA10-A   cctgcacaag tgaaggctag aaacatcatt agccctgtga tgggggtaat tgggttcagc
BAV BA16-L   cctgcacaag tgaaagctag aaacatcatt agccctgtga tgggggtaat tgggtttagc
BAV BA02-C   cctgcacaag tgaaagctag aaacatcatt agccctgtga tgggggtaat tgggtttagc
BAV BA17-S   cctgcacaag tgaaagctag aaacatcatt agccctgtga tgggggtaat tgggtttagc
BAV BA11-A   cctgcacaag tgaaagctag aaacatcatt agccctgtga tgggggtaat tgggtttagc
BAV BA02-C   cctgcacaag tgaaagctag aaacatcatc agccctgtga tgggggtaat tgggtttagc
BAV BA02-C   cctgcacaag tgaaagctag aaacatcatt agccctgtga tgggggtaat tgggtttagc
BAV BA02-C   cctgcacaag tgaaagctag aaacatcatt agccctgtga tgggggtaat tgggtttagc
BAV Hu3969   cctgcacaag tgaaagctag gaacatcatt agtcctgtga tgggggtaat tgggtttagc
ANDV CHI-9   ccagcacagg tcaaagcccg aaatataata agtcctgtaa tgggagtaat tggatttggc
ANDV AREB1   ccagcacagg tcaaagctcg aaacataata agccctgtaa tgggagtgat tggatttggc
ANDV Epuye   ccagcacagg tcaaagctcg aaacataata agccctgtaa tgggagtgat tggatttggc
ANDV Epili   ccagcacagg tcaaagctcg aaacataata agccctgtaa tgggagtgat tggatttggc
ANDV NRC-2   ccagcacagg ttaaagctcg aaatataata agtcctgtga tgggagtaat tggatttgga
ANDV NRC-4   ccagcacagg tcaaagctcg aaatataata agtcctgtga tgggagtaat tggatttggc
ANDV NRC-6   ccagcacagg tcaaagctcg aaacataata agccctgtaa tgggagtgat tggatttggc

             ....|....| ....|....| ....|....| ....|....| ....|....| ....|....|
                  725        735        745        755        765        775
SNV NM H10   tttttgtga aagattggat ggaaaggatt gatgacttcc tggctgcacg ttgcccattt
RIOMV HTN-   cactttgtga aggactggat ggaaaggatt gacaactttc taggagaaga ttgcccattt
LANV 510B    cactttgtga aggactggat ggaaaggatt gacaactttc taggagaaga ttgcccattt
CHOV 588     tttttgtaa aggattggat ggatcgtatt gataatttct tatctgctga gtgtccattt
JABV AKM96   tttttgtta aggattggat ggatcggata gaagactttt tggctgcaga gtgccctttt
MAPV HV-97   ttctttgtga aggactggat ggataggatt gaagaattcc ttggtgcaga atgtccattc
JUQV oln19   tttttgtca aagattggat ggaaaggatt gaaaatttct tagctgctga atgcccattt
JUQV LH      ttctttgtaa aggactggat ggaaagaatt gaaaacttcc tggctgctga atgcccgttt
PERV 14403   ttcttcgtca aggactggat ggaaagaatt gaagatttct tagctgcaga gtgtccgttt
MACV 13796   ttctttgtga aggattggat ggaacggata gagggattct tggcagcgga atgccctttc
ORNV O1229   ttctttgtga aggattggat ggaaaggatt gaggatttcc tggcagctga atgcccattc
ORNV AND N   ttctttgtga aggattggat ggaaaggatt gaggatttct tggcagctga atgcccattc
LECV BMJ-N   ttctttgtaa aggactggat ggaaaggatt gaagacttct tggcagctga atgccctttc
LECV BMJ-O   ttctttgtaa aagattggat ggaaaggata gaagactttt tggcagctga atgtcctttc
LECV Plata   ttctttgtga aggactggat ggaaaggata gaagatttct tggcagctga atgtcctttc
LECV CO22-   ttctttgtga aggattggat ggaaaggata gaagatttct tggcagctga atgtcctttc
LECV BA18-   ttctttgtga aggattggat ggaaaggata gaagatttct tggcagctga atgtcctttc
LECV BA18-   ttctttgtga aggattggat ggaaaggata gaggatttct tggcagctga atgtcctttc
LECV ER19-   ttctttgtga aagattggat ggaaaggata gaagatttct tggcagctga atgtcctttc
LECV 22819   ttctttgtga aggattggat ggaaaggata gaagatttcc tggcagctga atgtcctttc
BAV BA10-A   ttctttgtta aagactggat ggaaaggata gaagattttc tggcagcgga atgcccattt
BAV BA16-L   ttctttgtta aagactggat ggaaaggata gaagattttc tggcagctga atgcccattt
BAV BA02-C   ttctttgtta aagactggat ggaaaggata gaagattttc tggcggctga atgcccattt
BAV BA17-S   ttctttgtta aagactggat ggaaaggata gaagattttc tggcagctga atgcccattt
BAV BA11-A   ttctttgtta aagactggat ggaaaggata gaagattttc tggcagctga atgcccattt
BAV BA02-C   ttctttgtta aggactggat ggaaaggata gaagattttc tggcagctga atgcccattt
BAV BA02-C   ttctttgtta aggactggat ggaaaggata gaagattttc tggcagctga atgcccattt
BAV BA02-C   ttctttgtta aggactggat ggaaaggata gaagattttc tggcagctga atgcccattt
BAV Hu3969   ttctttgtta aagactggat ggaaaggatt gaagattttc tggcagctga atgcccattt
ANDV CHI-9   ttctttgtaa aggattggat ggatcggata gaagagtttc tggctgcaga gtgtccattc
ANDV AREB1   ttctttgtga aggactggat ggatcggata gaggaattcc tggctgcaga gtgtccattc
ANDV Epuye   ttctttgtga aggactggat ggatcggata gaggaattcc tggctgcaga gtgtccattc
ANDV Epili   ttctttgtga aggactggat ggatcggata gaggaattcc tggctgcaga gtgtccattc
ANDV NRC-2   tttttgtaa aggactggat ggatcggata gaggaattcc tggctgcaga gtgtccattc
ANDV NRC-4   ttctttgtaa aggactggat ggatcggata gaggaattcc tggctgcaga gtgtccattc
ANDV NRC-6   ttctttgtga aggactggat ggatcggata gaggaattcc tggctgcaga gtgtccattc

             ....|....| ....|....| ....|....| ....|....| ....|....| ....|....|
                  785        795        805        815        825        835
SNV NM H10   ctgcctgagc agaaagaccc tagagatgct gcattggcaa ctaatagagc ctattttata
```

```
RIOMV HTN-   cttccaaagg ctagggttca atcagaggca ttcatgtcaa cgaatagggc atattttcta
LANV 510B    cttccaaagg ctagggttca atcagaggca ttcatgtcaa cgaatagggc atattttcta
CHOV 588     ttgcagaagc caaaagttcc atctgaggca tttacaacca caaatcgggc atattttctg
JABV AKM96   ctgccaaaac caaagaatca ggcagatgca ttcatgggga caaacagagc ttattttatt
MAPV HV-97   ctccccaaac caaaatccca gtcagagaat ttcatgtcaa caaatagggc ttatctaatt
JUQV oln19   cttccaaaac caaaggtcgc atctgaggca ttcatggcta caaacaaggc atacttctta
JUQV LH      cttccaaaac cgaaggttgc atctgaggca ttcatggcaa caaataaggc atacttttta
PERV 14403   ctacctaagc ctaaaaccac ctctgaggca ttcatgtcaa ctaataaggc ttatttctta
MACV 13796   ttacctaagc ccaaaacagc tgctgagaca ttcatggcca ccaataaggc atactttttt
ORNV 01229   ttacctaaac ccaaggcatt gtctgaagca ttcatggcta ctaataaagc ttatttcctg
ORNV AND N   ttacctaaac ccaaggcatt gtctgaagca ttcatggcta ctaataaagc ttatttcctg
LECV BMJ-N   ctgccgaagc caaaggctcc atctgaagca ttcatggcca ctaacaaggc ctatttttta
LECV BMJ-O   ctaccaaagc caaaggctcc atctgaagca ttcatggcca ctaacaaagc ctatttttta
LECV Plata   ttacccaaac ctaaggctcc atctgaagcg ttcatggcca ccaataaagc ctatttctta
LECV CO22-   ttacccaaac caaaggcacc atctgaagca ttcatggcca ccaataaagc ctacttcttg
LECV BA18-   ttacccaaac caaaggcacc atctgaagca ttcatggcca ccaataaagc ctacttcttg
LECV BA18-   ttacccaaac caaaggcacc atctgaagca ttcatggcca ccaataaagc ctacttcttg
LECV ER19-   ttacccaaac caaaggcacc atctgaagca ttcatggcca ccaataaagc ctacttcttg
LECV 22819   ttacccaaac caaaggcacc atctgaagca ttcatggcca ccaataaagc ctacttcttg
BAV BA10-A   ttgcccaagc ctaaagctcc atctgaggca tttatggcta ccaacaaagc atatttcttg
BAV BA16-L   ttgcccaagc ctaaagctcc atctgaggca tttatggcta ccaacaaagc gtatttcttg
BAV BA02-C   ttgcccaagc ctaaagctcc atctgaggca tttatggcta ccaacaaagc gtatttcttg
BAV BA17-S   ttgcccaagc ctaaagctcc atctgaggca tttatggcta ccaacaaagc gtatttcttg
BAV BA11-A   ttgcccaagc ctaaagctcc atctgaggca tttatggcta ccaacaaagc gtatttcttg
BAV BA02-C   ttgcccaagc ctaaagctcc atctgaggca tttatggcta ccaacaaagc atatttcttg
BAV BA02-C   ttgcccaagc ctaaagctcc atctgaggca tttatggcta ccaacaaagc atatttcttg
BAV BA02-C   ttgcccaagc ctaaagctcc atctgaggca tttatggcta ccaacaaagc atatttcttg
BAV Hu3969   ttgcccaagc ctaaagctcc atctgaggca tttatggcta ccaacaaagc gtatttcttg
ANDV CHI-9   ttacctaagc caaaggtcgc ctcagaagcc ttcatgtcta ccaataagat gtattttctg
ANDV AREB1   ttgcctaagc caaaggttgc ctcagaagcc ttcatgtcta ccaacaagat gtatttcctg
ANDV Epuye   ttgcctaagc caaaggttgc ctcagaagcc ttcatgtcta ccaacaagat gtatttcctg
ANDV Epili   ttgcctaagc caaaggttgc ctcagaagcc ttcatgtcta ccaacaagat gtatttcctg
ANDV NRC-2   ttgcctaagc caaaggttgc ctcagaagcc ttcatgtcta ccaacaagat gtatttcctg
ANDV NRC-4   ctgcctaagc caaaggttgc ctcagaagcc ttcatgtcta ccaacaagat gtatttcctg
ANDV NRC-6   ttgcctaagc caaaggttgc ctcagaagcc ttcatgtcta ccaacaagat gtatttcctg

             ....|....| ....|....| ....|....| ....|....| ....|....| ....|....|
                   845        855        865        875        885        895
SNV NM H10   acacgtcaat tacaggttga tgagtcaaag gttagtgata ttgaggacct gattgctgat
RIOMV HTN-   aacaggcaga ggcaagtgga tgagtcgaga atacaggaca ttgtggattt aatacaagca
LANV 510B    aacaggcaga ggcaagtgga tgagtcgaga atacaggaca ttgtggattt aatacaagca
CHOV 588     gcaaggcaaa gtcaggtgag agagtctatt cttcctgaca ttgaagactt aattgataca
JABV AKM96   aacaggcaaa accaagttga ggagtcgaag atccctgaca ttgttgactt aatcgatgcg
MAPV HV-97   aacagacaac agcaagttcg ggaatcccag gtggctgaaa ttgtggacct catccagaca
JUQV oln19   aataggcagc gtcaagttaa tgattcaaag gtccaagaca tcgtagacct tatcgagcat
JUQV LH      aaccggcagc gccaagtcaa tgactcaaag gtccaggaca ttgtggacct tattgaccat
PERV 14403   aacaggcaga gacaggttga tgaatctcaa gtcccagaca tagcagaact gatagaacag
MACV 13796   aacaggcaac gccaggttga tgagtctcaa gtcccagata ttgttgaact tattgaccag
ORNV 01229   aataggcaac ggcaggtgaa tgattcaaaa gtccaagata tagttgacct cattgaccat
ORNV AND N   aataggcaac ggcaggtgaa tgattcaaaa gtccaagata tagttgacct cattgaccat
LECV BMJ-N   aataggcaac ggcaagtaaa tgagtcaaaa gtccaagata tagttgatct cattgaccat
LECV BMJ-O   aatagacaaa ggcaagtgaa tgagtcaaaa gtccaggata tagttgacct cattgaccat
LECV Plata   aacaggcaaa gacaggtcaa cgagtcaaaa gtccaggata tagttgacct catcgatcat
LECV CO22-   aacagacaac gacaggttaa tgagtcaaaa gttcaagaca tagttgacct cattgaccat
LECV BA18-   aacagacaac gacaggttaa tgagtcaaaa gttcaggaca tagttgacct cattgatcac
LECV BA18-   aacagacaac gacaggttaa tgagtcaaaa gttcaggaca tagttgacct cattgatcac
LECV ER19-   aacagacaac gacaggttaa tgagtcaaaa gttcaggaca tagttgacct cattgatcac
LECV 22819   aacagacaac gacaggttaa tgagtcaaaa gttcaggaaa tagttgacct cattgatcac
BAV BA10-A   aacaggcaga ggcaagtcaa cgaatctaag gtccaagata ttgtggactt aattgatcat
BAV BA16-L   aacaggcaga ggcaagtcaa cgaatctaag gtccaagata ttgtggactt aattgatcat
BAV BA02-C   aacaggcaga ggcaagtcaa cgaatctaag gtccaagata ttgtggactt aattgatcat
BAV BA17-S   aacaggcaga ggcaagtcaa cgaatctaag gtccaagata ttgtggactt aattgatcat
BAV BA11-A   aacaggcaga ggcaagtcaa cgaatctaag gtccaagata ttgtggactt aattgatcat
BAV BA02-C   aacaggcaga ggcaagtcaa cgaatctaag gtccaagata tcgtggactt aattgatcat
```

```
BAV BA02-C    aacaggcaga ggcaagtcaa cgaatctaag gtccaagata tcgtggactt aattgatcat
BAV BA02-C    aacaggcaga ggcaagtcaa cgaatctaag gtccaagata tcgtggactt aattgatcat
BAV Hu3969    aacaggcaga ggcaagtcaa cgaatctaaa gtccaagata ttgtagactt aatcgatcat
ANDV CHI-9    aacagacaga gacaagtcaa tgaatctaag gttcaagata ttatcgattt gatagaccat
ANDV AREB1    aacagacaga gacaagtcaa tgaatctaag gttcaagaca ttattgattt gatagatcat
ANDV Epuye    aacagacaga gacaagtcaa tgaatctaag gttcaagaca ttattgattt gatagatcat
ANDV Epili    aacagacaga gacaagtcaa tgaatctaag gttcaagaca ttattgattt gatagatcat
ANDV NRC-2    aacagacaaa gacaggtcaa tgaatctaag gttcaagaca ttattgattt aatagatcat
ANDV NRC-4    aacagacaaa gacaagtcaa tgaatctaag gttcaagaca tcattgattt aatagatcat
ANDV NRC-6    aacaggcaga gacaagtcaa tgaatctaag gttcaagaca ttattgattt gatagatcat

              ....|....| ....|....| ....|....| ....|....| ....|....| ....|....|
                   905        915        925        935        945        955
SNV NM H10    gcaagggctg agtctgccac tatattcgca gatattgcta ctcctcattc agtttgggtc
RIOMV HTN-    gcagaggcaa gttcagctac actattcaat gacattgcaa caccacattc tgcatgggtt
LANV 510B     gcagaggcaa gttcagctac actattcaat gacattgcaa caccacattc tgcatgggtt
CHOV 588      gctgagtctg aaggtgccac cctatttaaa gacattgctg tccctcactc tgtgtgggtt
JABV AKM96    gcagaagcag agggggcaac attgtctcga gacatagcag taccacattc tgcatggatt
MAPV HV-97    gctgaggcag aatctgccac attattccat gatattgcca gcccccattc tgtctgggtt
JUQV oln19    gcagagatcg agtctgcaac attgtttact gacattgcaa caccacattc tgtttgggtg
JUQV LH       gcagaaattg aatctgcaac attgtttact gacattgcaa caccacattc tgtatgggta
PERV 14403    gcggaatctg agtcagcaac tttattcaca gaaattgcaa gccctcattc agtatgggta
MACV 13796    gctgagtctg agtctgccac cctgtttaca gaaatagcta gcccccattc agtgtgggtg
ORNV O1229    gcagagattg aatcagcaac acttttcaca gacattgcaa ctccccattc agtttgggta
ORNV AND N    gcagagattg aatcagcaac acttttcaca gacattgcaa ctcctcattc agtttgggta
LECV BMJ-N    gcagagaccg agtcagcaac actgttcaca gatattgcta ctccacactc agtgtgggta
LECV BMJ-O    gcggagactg agtcagcaac attgtttacg gatattgcta ctccacactc agtgtgggtg
LECV Plata    gcagagactg agtcagcaac attgttcaca gatattgcta ctccacactc agtgtgggtg
LECV CO22-    gcagagaccg agtcagcaac actgtttaca gatattgcaa ctccacactc agtgtgggtg
LECV BA18-    gcagagactg agtcagcaac actgtttaca gatattgcaa ctccacactc agtgtgggtg
LECV BA18-    gcagagactg agtcagcaac actgtttaca gatattgcaa ctccacactc agtgtgggta
LECV ER19-    gcagagactg agtcagcaac actgtttaca gatattgcaa ccccacactc agtgtgggtg
LECV 22819    gcagagactg agtcagcaac attgtttaca gatattgcaa ctccacactc agtatgggtg
BAV BA10-A    gcagagactg aatcggcaac actgtttaca gatattgcca ccccacactc agtgtgggta
BAV BA16-L    gcagagactg aatcggcaac actgtttaca gatattgcca ccccacactc agtgtgggta
BAV BA02-C    gcagagactg aatcggcaac cctgtttaca gatattgcca ccccacactc agtgtgggta
BAV BA17-S    gcagagactg aatcggcaac actgtttaca gatattgcca ccccacactc agtgtgggta
BAV BA11-A    gcagagactg aatcggcaac actatttaca gatattgcca ccccacactc agtgtgggta
BAV BA02-C    gcagagactg aatcggcaac actgtttaca gatattgcca ccccacactc agtgtgggta
BAV BA02-C    gcagagactg aatcggcaac actgtttaca gatattgcca ccccacactc agtgtgggta
BAV BA02-C    gcagagactg aatcggcaac actgtttaca gatattgcca ccccacactc agtgtgggta
BAV Hu3969    gcagagactg aatcggcaac actgtttaca gatattgcca ccccacactc agtgtgggta
ANDV CHI-9    gctgagaccg agtctgctac cttgtttaca gagattgcaa caccccattc agtctgggtg
ANDV AREB1    gctgaaactg aatctgccac cttgtttacg gaaattgcaa caccccattc agtctgggtg
ANDV Epuye    gctgaaactg agtctgctac cttgtttacg gaaattgcaa caccccattc agtctgggtg
ANDV Epili    gctgaaactg agtctgctac cttgtttacg gaaattgcaa caccccattc agtctgggtg
ANDV NRC-2    gctgaaactg agtctgctac cttgtttaca gaaattgcaa caccccattc agtctgggtg
ANDV NRC-4    gctgaaactg agtctgctac cttgtttaca gaaattgcaa caccccattc agtctgggtg
ANDV NRC-6    gctgaaactg agtctgctac cttgtttacg gaaattgcaa caccccattc agtctgggtg

              ....|....| ....|....| ....|....| ....|....| ....|....| ....|....|
                   965        975        985        995       1005       1015
SNV NM H10    tttgcatgtg ctccagatcg ttgtccacct acagcattat atgtggccgg gatgccggaa
RIOMV HTN-    tttgcatgtg ccccagatag gtgtcctcca actgcattat atgttgcagg tgtcccagaa
LANV 510B     tttgcatgtg ccccagatag gtgtcctcca actgcattat atgttgcagg tgtcccagaa
CHOV 588      tttgcctgtg caccagaccg gtgtccacca acagcactat atattgctgg gatgcctgag
JABV AKM96    tttgcatgcg caccagatag gtgcccacca actgcattat atgttgcagg aatcccagaa
MAPV HV-97    tttgcatgtg cacctgatcg atgccctcca actgctttat atgttgcagg cgttcctgag
JUQV oln19    tttgcatgtg cacctgatag atgcccacca acggcattgt atgtagcagg agtcccatat
JUQV LH       tttgcatgtg cacctgatag atgcccaccg acagcattgt atgtagcagg agttccagag
PERV 14403    tttgcatgtg caccagatag atgcccgcct acagccctgt atgttgcagg agtaccagaa
MACV 13796    tttgcatgtg cacctgatag gtgtccaccg acagcattat atgtggcagg agtgccagag
ORNV O1229    ttcgcatgtg caccagatcg ctgcccaccg acagccttat atgttgcagg ggttcctgag
ORNV AND N    ttcgcatgtg caccagatcg ctgcccaccg acagccttat atgttgcagg ggttcctgag
```

```
LECV BMJ-N   tttgcatgtg caccagatcg ctgtccacca acagctttat atgttgctgg tgttccagaa
LECV BMJ-O   tttgcatgtg caccagatcg ttgtccacca acagccttat atgtcgccgg tgttccagaa
LECV Plata   tttgcatgtg caccagatcg ttgcccaccg acagccttat atgttgctgg tgttccagaa
LECV CO22-   tttgcatgcg caccagatcg ttgtccacca acagccctat atgttgctgg tgttccagaa
LECV BA18-   tttgcatgtg caccagatcg ttgtccacca acagccttat atgttgccgg tgttccagaa
LECV BA18-   tttgcatgtg caccagatcg ttgtccacca acagccttat atgttgccgg tgttccagaa
LECV ER19-   tttgcatgtg caccagatcg ttgtccacca acagctctat atgttgcagg tgttccagaa
LECV 22819   tttgcatgtg caccagatcg ttgcccacca acagccttat atgttgccgg tgttccagaa
BAV BA10-A   tttgcatgtg caccggatcg ttgcccaccg acagcattgt atgttgccgg ggtccctgag
BAV BA16-L   tttgcatgtg caccggatcg ttgcccacca acaacattgt atgttgccgg ggtccctgag
BAV BA02-C   tttgcatgtg caccggatcg ttgcccaccg acagcattgt atgttgccgg ggtccctgag
BAV BA17-S   tttgcatgtg caccggatcg ttgcccaccg acagcattgt atgttgccgg ggtccctgag
BAV BA11-A   tttgcatgtg caccggatcg ttgcccaccg acagcattgt atgttgccgg ggtccctgag
BAV BA02-C   tttgcatgtg caccggatcg ttgcccaccg acagcattgt atgttgccgg ggtccctgag
BAV BA02-C   tttgcatgtg caccggatcg ttgcccaccg acagcattgt atgttgccgg ggtccctgag
BAV BA02-C   tttgcatgtg caccggatcg ttgcccaccg acagcattgt atgttgccgg ggtccctgag
BAV Hu3969   tttgcatgtg caccagatcg ttgcccacca acagcattgt atgttgccgg ggtccctgag
ANDV CHI-9   tttgcatgtg cacctgaccg gtgccctcca actgcattgt atgttgcagg ggtaccggaa
ANDV AREB1   tttgcatgtg cgcctgaccg gtgccctcca actgcattgt atgttgcagg ggtaccagaa
ANDV Epuye   tttgcatgtg cgcctgaccg gtgccctcca actgcattgt atgtcgcagg ggtaccagaa
ANDV Epili   tttgcatgtg cgcctgaccg gtgccctccg actgcattgt atgttgcagg ggtaccagaa
ANDV NRC-2   tttgcatgtg cgcctgaccg gtgccctccg actgcattgt atgttgcagg ggtaccagaa
ANDV NRC-4   tttgcatgtg cgcctgaccg gtgccctccg actgcattgt atgttgcagg ggtaccagaa
ANDV NRC-6   tttgcatgtg cgcctgaccg gtgccctcca actgcattgt atgttgcagg ggtaccagaa

             ....|....| ....|....| ....|....| ....|....| ....|....| ....|....|
                   1025       1035       1045       1055       1065       1075
SNV NM H10   ctgggtgcat tttttgctat tctccaggat atgaggaaca ccataatggc atccaaatct
RIOMV HTN-   ctcggggcat tctttgccat actccaggat atgagaaata caatcatggc atctaaatct
LANV 510B    ctcggggcat tctttgccat actccaggat atgagaaata caatcatggc atctaaatct
CHOV 588     cttggagctt tcttcgcaat acttcaagat atgagaaaca ccatcatggc atcaaaatct
JABV AKM96   ttgggtgctt tcttctcaat ccttcaagat atgagaaaca ccattatggc atcaaaatca
MAPV HV-97   cttggggcat tcttctccat cctacaggac atgagaaata ccatcatggc ctcaaagtct
JUQV oln19   ttgggagcat tctttgccat actccaggat atgcgcaata ccatcatggc ttcaaagtca
JUQV LH      ctaggtgcat tttttgctat acttcaggat atgcgcaaca ccatcatggc ttcaaagtca
PERV 14403   cttggggcct tttttgctat actacaagac atgaggaaca caataatggc atcaaagtcg
MACV 13796   ctaggtgcct tttttgccat actacaagat atgagaaata ccattatggc atcaaagtct
ORNV Ol229   ctaggggctt tctttgccat ccttcaggac atgcgaaata caatcatggc atcaaagtct
ORNV AND N   ctaggggctt tctttgccat ccttcaggac atgcgaaata caatcatggc atcaaagtct
LECV BMJ-N   ttgggtgcat tttttgccat cttacaggac atgcgaaaca caatcatggc ctcaaaatct
LECV BMJ-O   ttgggtgcat tctttgccat tttgcaggac atgcgaaaca caattatggc ttcaaagtct
LECV Plata   ttgggtgcat tctttgccat cctacaggat atgcggaata caatcatggc ttcaaagtct
LECV CO22-   ctgggtgcat tctttgctat cctacaggat atgagaaaca cgatcatggc atcaaagtct
LECV BA18-   ctgggtgcat tttttgctat tctacaggat atgcgaaata cgatcatggc atcaaagtct
LECV BA18-   ctgggtgcat tttttgctat tctacaggat atgcgaaata cgatcatggc atcaaagtct
LECV ER19-   ctgggtgcat tttttgctat tctacaggat atgcggaata caatcatggc atcaaagtct
LECV 22819   ctgggtgcat tttttgctat tctacaggat atgcggaata caatcatggc atcaaagtct
BAV BA10-A   ctaggtgcat tctttgctat tttgcaagac atgcggaata caatcatggc atcaaaatct
BAV BA16-L   ctaggtgcat tttttgctat tttgcaagac atgcggaata caatcatggc atcaaaatct
BAV BA02-C   ctaggtgcat tttttgctat tttgcaagac atgcggaata caatcatggc atcaaaatct
BAV BA17-S   ctaggtgcat tttttgctat tttgcaagac atgcggaata caatcatggc atcaaaatct
BAV BA11-A   ctaggtgcat tttttgctat tttgcaagac atgcggaata caatcatggc atcaaaatct
BAV BA02-C   ctaggtgcat tttttgctat tttgcaagac atgcggaata caatcatggc atcaaaatct
BAV BA02-C   ctaggtgcat tttttgctat tttgcaagac atgcggaata caatcatggc atcaaaatct
BAV BA02-C   ctaggtgcat tttttgctat tttgcaagac atgcggaata caatcatggc atcaaaatct
BAV Hu3969   ctaggtgcat tctttgctat tttgcaagac atgcggaata caatcatggc atcaaaatct
ANDV CHI-9   cttggtgcat ttttttctat ccttcaggac atgcgtaata ccatcatggc atctaaatct
ANDV AREB1   cttggtgcat tcttttccat tcttcaggac atgcgtaata ccatcatggc atctaaatct
ANDV Epuye   cttggtgcat tcttttccat tcttcaggac atgcgtaata ccatcatggc atctaaatct
ANDV Epili   cttggtgcat tcttttccat tcttcaggac atgcgtaata ccattatggc atctaagtct
ANDV NRC-2   cttggtgcat ttttttccat ccttcaggac atgcgtaata ccatcatggc atccaaatct
ANDV NRC-4   cttggtgcat tcttttccat tcttcaggat atgcgtaata ccatcatggc atccaaatct
ANDV NRC-6   cttggtgcat tcttttccat tcttcaggac atgcgtaata ccatcatggc atctaaatct
```

```
             ....|....| ....|....| ....|....| ....|....| ....|....| ....|....|
                  1085       1095       1105       1115       1125       1135
SNV NM H10   gtggggacat ctgaagagaa attgaagaag aaatcagcat tctaccagtc atacttaaga
RIOMV HTN-   gttggaacag cagaagaaaa gttgaagaag aaatctgcct tctaccagtc atatttacgg
LANV 510B    gttggaacag cagaagaaaa gttgaagaag aaatctgcct tctaccagtc atatttacgg
CHOV 588     gtgggaactg ctgaggaaaa actgaagaag aaatctgcat tctatcagtc atatcttcga
JABV AKM96   gtcgggacag ctgaagaaaa acttaagaaa aaatctgcat tctaccagtc ttatttaagg
MAPV HV-97   gtaggcactt ctgaagagaa actgaagaaa aaatcagcct tttaccaatc atatctccgc
JUQV oln19   gttggcactg ctgaagaaaa gctgaagaag aagtctgcat tctaccaatc ttatctaaga
JUQV LH      gtcggcactg ctgaggagaa actcaagaaa aagtctgcat tctatcaatc ttacttgaga
PERV 14403   gtcgggactg cagaagagaa gctcaagaag aagtctgctt tctaccagtc atacttgcgg
MACV 13796   gtgggaacag ctgaggagaa gttaaagaag aaatcagcat tctatcaatc ttatttgaga
ORNV O1229   gtaggaacag ctgaagaaaa actcaagaag aagtctgcat tctaccagtc atacctaagg
ORNV AND N   gtaggaacag ctgaagaaaa actcaagaag aagtctgcat tctaccagtc atacctaagg
LECV BMJ-N   gtagggacag ctgaagaaaa gctcaagaag aagtctgctt tctaccagtc atacttaaga
LECV BMJ-O   gttgggacag ctgaagaaaa actcaagaaa aaatctgcct tctaccagtc atacttgaga
LECV Plata   gtaggaacag ctgaagaaaa attgaaaaag aaatctgctt tctaccagtc atacttaaga
LECV CO22-   gtaggaacag ctgaagagaa gctgaagaaa aaatctgcct tctaccagtc atacttgaga
LECV BA18-   gtaggaacag ctgaagagaa gctgaagaaa aaatctgcct tctaccagtc atacttgagg
LECV BA18-   gtaggaacag ctgaagagaa gctgaagaaa aaatctgcct tctaccagtc atacttgagg
LECV ER19-   gtaggaacag ctgaagagaa gctgaagaaa aaatctgcct tctaccagtc atacttaagg
LECV 22819   gtaggaacag ctgaagagaa gctgaagaaa aaatctgcct tctaccagtc atacttgagg
BAV BA10-A   gtgggaacag ctgaagaaaa gcttaagaag aaatctgcct tctaccagtc ttacctcagg
BAV BA16-L   gtgggaacag ctgaagaaaa gcttaagaag aaatctgcct tctaccagtc ttacctcagg
BAV BA02-C   gtgggaacag ctgaagaaaa gcttaagaag aaatctgcct tctaccagtc ttacctcagg
BAV BA17-S   gtgggaacag ccgaagaaaa gcttaagaag aaatctgcct tctaccagtc ttacctcagg
BAV BA11-A   gtgggaacag ccgaggaaaa gcttaagaag aaatctgcct tctaccagtc ttacctcagg
BAV BA02-C   gtgggaacag ccgaagaaaa gcttaagaag aaatctgcct tctaccagtc ttacctcagg
BAV BA02-C   gtgggaacag ccgaagaaaa gcttaagaag aaatctgcct tctaccagtc ttacctcagg
BAV BA02-C   gtgggaacag ccgaagaaaa gcttaagaag aaatctgcct tctaccagtc ttacctcagg
BAV Hu3969   gtgggaacag ctgaagaaaa gcttaagaag aaatctgcct tctaccagtc ttacctcagg
ANDV CHI-9   gtagggactg cagaagagaa gctaaagaaa aaatctgcct tctaccaatc atacctaaga
ANDV AREB1   gtagggactg cagaagagaa gctaaagaag aaatctgcct tctatcaatc ttacctgaga
ANDV Epuye   gtagggactg cagaagagaa gctaaagaag aaatctgcct tctatcaatc ttacctgaga
ANDV Epili   gtagggactg cagaagagaa gctaaagaag aaatctgcct tctatcaatc ttacctgaga
ANDV NRC-2   gtggggactg cagaagagaa gctgaagaag aaatctgcct tctatcaatc ttacctaaga
ANDV NRC-4   gtggggactg cagaagagaa gctgaagaag aaatccgctt tctatcaatc ttacctaaga
ANDV NRC-6   gtagggactg cagaagagaa gctaaagaag aaatctgcct tctatcaatc ttacctgaga

             ....|....| ....|....| ....|....| ....|....| ....|....| ....|....|
                  1145       1155       1165       1175       1185       1195
SNV NM H10   cgtactcagt caatgggaat tcaactggac cagaagataa tcatcttata catgagccat
RIOMV HTN-   agaactcaat caatggggat acagttagac caaaaaatca tcatacttta catgttgtct
LANV 510B    agaactcaat caatggggat acagttagac caaaaaatca tcatacttta catgttgtct
CHOV 588     aggacacagt ctatggggat tcaactagac caaaggataa tcatcctcta catgctccat
JABV AKM96   cgaacacagt caatgggaat acaactggat caaaagataa ttataatgta tatgcttcat
MAPV HV-97   agaacacaat caatgggaat ccagctcgac cagaagatca tcatcatgta catgctgcac
JUQV oln19   agaactcaat ctatggggat acaactggat caaaaaatta taattatgta catgctattc
JUQV LH      agaactcaat ctatggggat acaactggat cagaagatta taattatgta tatgttattc
PERV 14403   agaacccagt caatggggat tcagttggat cagaagatta taatcctcta tatgttgtca
MACV 13796   cgaacacagt caatggggat tcaactggac cagaagatca tcatcctgta tatgttgtct
ORNV O1229   cggacacaat ctatgggaat ccaactggac caaaagatca taatccttta catgctatcc
ORNV AND N   cggacacaat atatgggaat ccaactggac caaaagatca taatccttta catgctatcc
LECV BMJ-N   aggacacagt ccatggggat tcaactggac caaaagatca tcatcctcta catgttgtct
LECV BMJ-O   aggacacaat ctatggggat tcaactggac cagaagatca taattctcta catgttatca
LECV Plata   aggacgcaat ctatggggat tcaactggac caaaaaatca taatccttta catgctatca
LECV CO22-   aggacacaat ctatgggaat tcaactggat caaaagatca tcatcctcta tatgctatca
LECV BA18-   aggacacaat ctatgggaat tcaactggat caaaagatca taatcctcta tatgctatca
LECV BA18-   aggacacaat ctatgggaat tcaactggat caaaagatca taatcctcta tatgctatca
LECV ER19-   aggacacaat ctatgggaat tcaactggat caaaagatca taatcctcta tatgctatca
LECV 22819   aggacacaat ctatgggaat tcagctggat caaaagatca taatcctcta tatgctatca
BAV BA10-A   agaacgcaat caatgggaat tcaactggat cagaagatca taatcctcta catgctctca
BAV BA16-L   agaacgcaat caatgggaat ccaactggat cagaagatca taatactcta catgctctca
BAV BA02-C   agaacgcaat caatgggaat ccaactggat cagaagatca taatcctcta catgctctca
```

```
BAV BA17-S   agaacgcaat caatgggaat ccaactggat cagaagatca taatcctcta catgctctca
BAV BA11-A   agaacgcaat caatgggaat ccaactggat cagaagatca taatcctcta catgctctca
BAV BA02-C   agaacgcaat caatgggaat ccaattggat cagaaaatca taatcctcta catgctctca
BAV BA02-C   agaacgcaat caatgggaat ccaactggat cagaaaatca taatcctcta catgctctca
BAV BA02-C   agaacgcaat caatgggaat ccaactggat cagaagatca taatcctcta catgctctca
BAV Hu3969   agaacgcaat caatgggaat tcaactggat caaaagatca taatcctcta catgctctca
ANDV CHI-9   aggacacaat ctatgggaat ccaactggac cagaagatca taatccttta catgctatca
ANDV AREB1   aggacacaat ctatggggat tcaactggac caaaagatca taatcctcta catgctctca
ANDV Epuye   agaacacaat ctatggggat tcaactggac caaaagatca taatcctcta catgctttca
ANDV Epili   aggacacaat ctatggggat tcaactggac caaaagatca taatcctcta catgctctca
ANDV NRC-2   aggacacaat ctatgggaat tcaactggac cagaaaatca taattctcta catgctttca
ANDV NRC-4   aggacacaat ctatggggat tcaactggac cagaagatca taattcttta catgctttca
ANDV NRC-6   aggacacaat ctatggggat tcaactggac caaaagatca taatcctcta catgctctca

             ....|....| ....|....| ....|....| ....|....| ....|....| ....|....|
                 1205       1215       1225       1235       1245       1255
SNV NM H10   tggggaagag aggccgtgaa tcacttccat cttggagatg atatggatcc tgagcttagg
RIOMV HTN-   tggggaaagg aggcagttaa tcacttccac cttggtgatg acatggatcc tgagcttaga
LANV 510B    tggggaaagg aggcagttaa tcacttccac cttggtgatg acatggatcc tgagcttaga
CHOV 588     tggggaaaag aagcagtaaa ccactttcac cttggtgatg acatggatcc tgagctaagg
JABV AKM96   tggggaaagg aagctgtcaa ccatttccac cttggagatg acatggatcc tgaattgagg
MAPV HV-97   tggggaaagg aggctgtgaa tcatttccat ctaggggatg atatggatcc agagctgagg
JUQV oln19   tggggtaagg aagcagtcaa tcatttccat ttgggagatg atatggaccc agaactcagg
JUQV LH      tggggcaaag aggcagtcaa ccacttccat ctgggagatg atatggaccc agaactcagg
PERV 14403   tggggaaagg aggctgtcaa tcattttcat cttggtgatg atatggatcc tgagctcaga
MACV 13796   tggggtaagg aggctgtgaa ccatttccat cttggtgatg atatggatcc tgaattgaga
ORNV O1229   tggggcaagg aagcagtgaa ccatttccat cttggtgatg acatggatcc tgagctcaga
ORNV AND N   tggggcaagg aagcagtgaa ccatttccat cttggtgatg atatggatcc tgagctcaga
LECV BMJ-N   tggggaaaag aagcagtgaa ccacttccat cttggggatg atatggatcc tgagcttaga
LECV BMJ-O   tgggggaaag aagcagtgaa ccactttcat cttggtgatg atatggatcc tgagctcaga
LECV Plata   tggggaaagg aagcagtaaa ccacttccat cttggtgatg atatggatcc tgagcttaga
LECV CO22-   tgggggaaag aggcagtaaa tcactttcat ctcggtgatg acatggatcc tgagcttaga
LECV BA18-   tgggggaagg aggcagtaaa tcactttcat cttggtgatg atatggatcc tgagcttaga
LECV BA18-   tgggggaagg aggcagtgaa tcactttcat cttggtgatg atatggatcc tgagcttaga
LECV ER19-   tgggggaagg aggcagtaaa tcacttccat cttggtgatg atatggatcc tgagcttaga
LECV 22819   tgggggaagg aggcagtaaa tcactttcat cttggtgatg atatggatcc tgagcttaga
BAV BA10-A   tggggcaagg aagcagtgaa ccatttccat cttggcgatg acatggaccc tgaactgagg
BAV BA16-L   tggggcaagg aagcagtgaa ccatttccat cttggtgacg acatggaccc tgaactgagg
BAV BA02-C   tggggtaagg aagcagtgaa ccatttccat cttggtgacg acatggaccc tgaactgagg
BAV BA17-S   tggggcaagg aagcagtgaa ccatttccat cttggtgacg atatggaccc tgaactgagg
BAV BA11-A   tggggcaagg aagcagtgaa ccatttccac cttggtgacg acatggaccc tgaactgagg
BAV BA02-C   tggggcaagg aagcagtgaa ccatttccat cttggtgacg acatggaccc tgaactgagg
BAV BA02-C   tggggcaagg aagcagtgaa ccatttccat cttggtgacg acatggaccc tgaactgagg
BAV BA02-C   tggggcaagg aagcagtgaa ccatttccac cttggtgacg acatggaccc tgaactgagg
BAV Hu3969   tggggcaagg aagcagtgaa ccatttccat cttggtgatg acatggaccc tgaactgagg
ANDV CHI-9   tggggtaaag aagctgtgaa tcacttccat cttggtgatg atatggaccc tgaactcagg
ANDV AREB1   tggggtaaag aagctgtgaa tcatttccat cttggtgatg atatggaccc tgaactaagg
ANDV Epuye   tggggtaaag aagctgtgaa tcatttccat cttggtgatg atatggaccc tgaactaagg
ANDV Epili   tggggtaaag aagctgtgaa tcatttccat cttggtgatg atatggaccc tgaactaagg
ANDV NRC-2   tggggtaaag aagctgtgaa tcatttccat cttggtgatg atatggaccc tgaactgagg
ANDV NRC-4   tggggtaaag aagctgtgaa tcatttccat cttggtgatg atatggaccc tgaactaagg
ANDV NRC-6   tggggtaaag aagctgtgaa tcatttccat cttggtgatg atatggaccc tgaattaagg

             ....|....| ....|....| ....|....| ....|....| ....|....| ....|....|
                 1265       1275       1285       1295       1305       1315
SNV NM H10   gaacttgccc agacccttgt agatatcaag gtcagggaaa tctctaacca agaaccactt
RIOMV HTN-   cagctggcac agactctcat tgatgcaaag gtcaaagaga tctctaatca ggaaccccta
LANV 510B    cagctggcac agactctcat tgatgcaaag gtcaaagaga tctctaatca ggaaccccta
CHOV 588     cagttagcac agtccttagt tgacactaaa gtgaaggaga tttcaaacca ggaaccttta
JABV AKM96   cagctagctc aatcattaat tgacagcaag gttaaggaga tttctaatca agatccctta
MAPV HV-97   cagctagcac agtcattgat tgacaccaag gtaaaagaga tttccaacca agagcctttg
JUQV oln19   cagcttgctc aatctctcat tgacactaag gtcaaggaga tctccaacca agagccatta
JUQV LH      cagcttgctc aatctcttat tgacactaag gtcaaggaga tttccaacca agagccatta
PERV 14403   caactggctc aagctttgat tgacacaaaa gtaaaggaga tttctaacca agagccattg
```

```
MACV 13796   caactagcac aggctttaat tgatactaaa gtgaaggaga tctcaaacca ggagcctttg
ORNV O1229   caacttgctc aatccttgat tgataccaag gttaaggaaa tttcaaacca agagccatta
ORNV AND N   caacttgctc aatccttgat tgataccaag gttaaggaaa tttcaaacca agagccatta
LECV BMJ-N   cagcttgctc agtctttgat tgatactaag gtgaaagaga tttccaatca agagccacta
LECV BMJ-O   cagctcgctc aatccttgat tgatactaag gtgaaggaga tttccaatca agagccatta
LECV Plata   cagctagctc agtctttgat cgatactaag gtgaaggaga tttctaatca agagccactg
LECV CO22-   cagcttgctc aatccttgat tgacactaag gtgaaggaga tttccaatca ggagccatta
LECV BA18-   cagcttgctc agtccctgat tgacactaag gtgaaggaga tttctaatca ggagccatta
LECV BA18-   cagcttgctc agtccttgat tgacactaag gtgaaggaga tttctaatca ggagccatta
LECV ER19-   cagcttgctc agtccttaat tgacaccaag gtgaaggaga tttccaatca ggagccgtta
LECV 22819   cagcttgctc agtccttgat tgacactaag gtgaaggaga tttccaatca ggagccatta
BAV BA10-A   cagcttgctc aatctttgat tgatactaag gtgaaggaga tttcaaatca agagccattg
BAV BA16-L   cagcttgctc aatctctgat tgatactaag gtgaaggaaa tttcaaatca agagccattg
BAV BA02-C   cagcttgctc aatctctgat tgatactaag gtgaaggaaa tttcaaatca agagccattg
BAV BA17-S   cagcttgctc aatctctgat tgatactaag gtgaaggaaa tttcaaatca agagccattg
BAV BA11-A   cagcttgctc aatctctgat tgatactaag gtgaaggaaa tttcaaatca agagccatta
BAV BA02-C   cagcttgctc aatctctgat cgatactaag gtgaaggaaa tttcaaatca agagccactg
BAV BA02-C   cagcttgctc aatctctgat cgatactaag gtgaaggaaa tttcaaatca agagccactg
BAV BA02-C   cagcttgctc aatctctgat cgatactaag gtgaaggaaa tttcaaatca agagccattg
BAV Hu3969   cagcttgctc aatctcttat tgacactaag gtgaaagaga tttcaaatca agagccattg
ANDV CHI-9   cagctagcac aatctctgat cgatactaag gtgaaggaga tctccaacca agagccactt
ANDV AREB1   cagctagcac aatccctgat tgacaccaag gtgaaggaga tctccaacca agagccacta
ANDV Epuye   cagctagcac aatccctgat cgacaccaag gtgaaggaga tctccaacca agagccacta
ANDV Epili   cagctagcac aatccctgat tgacaccaag gtgaaggaga tctccaacca agagccacta
ANDV NRC-2   cagctagctc aatccctgat tgacaccaag gtgaaggaga tctccaacca agagccactt
ANDV NRC-4   cagctagctc aatccctgat tgacaccaag gtgaaggaga tctccaacca agagccactt
ANDV NRC-6   cagctagcac aatccctgat tgacaccaag gtgaaggaga tctccaacca agagccacta

             ....|....| ....|....| ....|....| ....|....| ....|....| ....|....|
                 1325       1335       1345       1355       1365       1375
SNV NM H10   aaactttaag tgggcagtaa tcaacttata ttcaggg--- ----cattat tataaccagg
RIOMV HTN-   aaactctaag a--------- ---------- ---------- ----aggcag taggatcaat
LANV 510B    aaactctaag a--------- ---------- ---------- ----aggcag taggatcaat
CHOV 588     aagctttaaa tgtgga---- ---------- ---------- ----tggtta aagggctgat
JABV AKM96   aaactgtgag ctatta---- -----tgatg tctg------ ----tgactg aaaggatgat
MAPV HV-97   aagttgtgag tc-------- ---------- ---------- agactggtag taaagattga
JUQV oln19   aaactgtagg tgct------ ---------- ---------- tcaaaacggt gggaaggtaa
JUQV LH      aaactgtagg tgct------ ---------- ---------- ctacaattat gaaggtg-aa
PERV 14403   aaattgtaag tg-------- ---------- ---------- --------gat aagggcagta
MACV 13796   aagctgtaag tgtcaa---- -----gggtg taggg----- ----tggtgt ttagtcttaa
ORNV O1229   aagctgtaag agcc-a---- -----taatt ataagagcca tta-tgctat gagagcgaaa
ORNV AND N   aagctgtaag agcc-a---- -----taatt ataagagcca tta-tgctat gagagccaaa
LECV BMJ-N   aagctgtaag agccaa---- -----caact agtaaagcca tcactgccaa aagagccaat
LECV BMJ-O   aagctgtgag agttga---- ------aact atcaaagcca tcactgccat aggagccaat
LECV Plata   aagctgtaag agctca---- -----caact ttcagagcca tcactgctat aagagccaaa
LECV CO22-   aagctgtaag agctga---- -----aaaat ttaagagcca tcactgccat aagagccaga
LECV BA18-   aagctgtaag agctga---- -----taaat ttaagagcca tcactgccat gagagccaga
LECV BA18-   aagctgtaag agctga---- -----taaac ttaagagcca tcactgccat aagagccaga
LECV ER19-   aagctgtaag agctaa---- -----taaat ttaagagcca tcactgccat aagagccaga
LECV 22819   aagctgtaag agctag---- -----taaat ttaagagcca tcactgccat aagagccaga
BAV BA10-A   aagttgtaag agccaa---- -----caaca tgtagg---- ----tgctac agtaactgga
BAV BA16-L   aagctgtaag agccaa---- -----caaca tgtagg---- ----tgctac agtaactgga
BAV BA02-C   aagctgtaag agccaa---- -----cagca tgtagg---- ----tgctac agtaactgga
BAV BA17-S   aagctgtaag agccaa---- -----caaca tgtagg---- ----tgctac agtaactgga
BAV BA11-A   aagctgtaag agccaa---- -----caaca tgtagg---- ----tgctac agtaactgga
BAV BA02-C   aagctgtaag agccaa---- -----caaca tgtagg---- ----tgctac agtaactgga
BAV BA02-C   aagctgtaag agccaa---- -----caaca tgtagg---- ----tgctac agtaactgga
BAV BA02-C   aagctgtaag agccaa---- -----caaca tgtagg---- ----tgctac agtaactgga
BAV Hu3969   aagctgtaag agccaa---- -----caaca tgtagg---- ----tgctag agtaactgga
ANDV CHI-9   aagttgtagg tgct------ ---------- ---------- -taatgaaat catgattgaa
ANDV AREB1   aagttataaa tact------ ---------- ---------- -tgatgaaag tatgattgaa
ANDV Epuye   aagttgtaaa tact------ ---------- ---------- -taatgaaag tatgatggaa
ANDV Epili   aagttgtaaa tact------ ---------- ---------- -taatgaaag tatgattgaa
ANDV NRC-2   aagttgtaag tgct------ ---------- ---------- -taatgaaag gataattgaa
```

```
ANDV NRC-4    aagttgtaag tgct------ ---------- ---------- -tgatgaaag gatgattgaa
ANDV NRC-6    aagttgtaaa tact------ ---------- ---------- -taatgaaag tatgattgaa

              ....|....| ....|....| ....|....| ....|....| ....|....| ....|....|
                   1385       1395       1405       1415       1425       1435
SNV NM H10    ---------- -------gta atgggcacta atcagggttt attgcactag ggtgggttca
RIOMV HTN-    ---------- ---------g attgtggtat gtgagggctt tccgggct-- --tgtgcca-
LANV 510B     ---------- ---------g attgtggtat gtgagggctt tccgggct-- --tgtgcca-
CHOV 588      ---------- ---------- ---------- --gagggctt tctgggct-- --tgtgccaa
JABV AKM96    ---------- ---------- ---------- --agagggct cctggggc-- --tgtgccat
MAPV HV-97    ---------- ---------- ---------- ----gggctt ttcgggct-- --tgtgccaa
JUQV oln19    ---------- ---------- ---------- gtgaaggcgt ttcgggct-- --tgagccaa
JUQV LH       ---------- ---------- ---------- gtgaaggcgt ttcgggct-- --tgagccaa
PERV 14403    ---------- ---------- --tgatgctt ggacgg-ttt ttcgggct-- --tgtgccac
MACV 13796    ---------- ---------- ---------t gatgggtttt caggggct-- --tgtgccat
ORNV O1229    atagtttaca tcttgatggt tatatcagtc agacgg-ctt tccgggct-- --tgtgccac
ORNV AND N    atagtttaca tcttgatggt tatatcactc agatgg-ctt tccgggct-- --tgtgccac
LECV BMJ-N    accagtcaag atctatggat tatgttagtt agatggattt tctgggct-- --tgtgccaa
LECV BMJ-O    actagttgat atctaagaat caggttggtt agatggattt tctgggct-- --tgtgccaa
LECV Plata    --tagttaat atctggagat gctgttggtt aggtgggttt ttcgggct-- --tgtgccac
LECV CO22-    actagtcaat atctgaagat tatgttgatt ggtggatttt ccggggct-- --tgtgccac
LECV BA18-    actagtcaat atctgaaaat tatgttagtt gggtggattt tccgggct-- --tgtgccac
LECV BA18-    actagtcaat atctgaagat tatgttggtt gggtggattt tccgggct-- --tgtgccac
LECV ER19-    actagtcaat atctgaaggt tatgttggtt gggtggagtt tccgggct-- --tgtgccac
LECV 22819    actagtcaat atctgaagat tatgttggtt gggtggattt tccgggct-- --tgtgccac
BAV BA10-A    ---------- ---------- ----taggtt gggtggtctt tctgggct-- --tgagccac
BAV BA16-L    ---------- ---------- ----taggtt ggatggtctt tctgggct-- --tgagccac
BAV BA02-C    ---------- ---------- ----taggtt ggatggtctt tctgggct-- --tgagccac
BAV BA17-S    ---------- ---------- ----taggtt ggatggtctt tctgggct-- --tgagccac
BAV BA11-A    ---------- ---------- ----taggtt ggatggtctt tctgggct-- --tgagccac
BAV BA02-C    ---------- ---------- ----taggtt ggatggtctt tctgggct-- --tgagccac
BAV BA02-C    ---------- ---------- ----taggtt ggatggtctt tctgggct-- --tgagccac
BAV BA02-C    ---------- ---------- ----taggtt ggatggtctt tctgggct-- --tgagccac
BAV Hu3969    ---------- ---------- ----taggtt ggatggtctt tctgggct-- --tgagccac
ANDV CHI-9    ---------- ---------- ---------- -gaaagactt tccgggct-- --tgtgccac
ANDV AREB1    ---------- ---------- ---------- -gagagactt tccgggct-- --tgtgccac
ANDV Epuye    ---------- ---------- ---------- -gagagactt tccgggct-- --tgtgccac
ANDV Epili    ---------- ---------- ---------- -gagagactt tccgggct-- --tgtgccac
ANDV NRC-2    ---------- ---------- ---------- -gaaagactt tccgggct-- --tgtgccac
ANDV NRC-4    ---------- ---------- ---------- -gaaagactt tccgggct-- --tgtgccac
ANDV NRC-6    ---------- ---------- ---------- -gagagactt tccgggct-- --tgtgccac

              ....|....| ....|....| ....|....| ....|....| ....|....| ....|....|
                   1445       1455       1465       1475       1485       1495
SNV NM H10    agggccaatt atatcacagg gtatataatc tatattgttt agtgttattg ----------
RIOMV HTN-    acatttattc atctcagggc ---------- ----tattct gcactaatca atgggtggga
LANV 510B     acatttattc atctcagggc ---------- ----tattct gcactaatca atgggtggga
CHOV 588      ata-ttaatc atttcagggc ttt-----ac actaatcggg aatgtgacta ctatcag---
JABV AKM96    a---ttaatc atctcaatct ---------- ----atctct gacg------ ----------
MAPV HV-97    ttgtt----- -actcagggg ct-------- ----actgtc taattaattg cactgggttc
JUQV oln19    tcagttattc atctcaggat ct-------- ----ttcctt aatgtgacta ctaaggg-t-
JUQV LH       tcagttattc atctcaggat ct-------- ----ttcctt aatgtgacta ctaagggtt-
PERV 14403    acaattaatc atctcagggc ---------- -------ttt actgtgaata acagggttt-
MACV 13796    atgtttaatc atctcagggg c--------- -----tgtct actgtgatta acagggtg--
ORNV O1229    atatttaatc atctcaggaa ---------- -----ccttt aatgtgatta atacgggt--
ORNV AND N    atatttaatc atctcaggaa ---------- -----ccttt aatgtgatta atacgggt--
LECV BMJ-N    ttatttaatc atctcaggac ---------- -----tctct aatgtgatta atacgggt--
LECV BMJ-O    tcatttaatc atctcaggac ---------- -----tctct aatgtgatta atacgggt--
LECV Plata    atatttaatc atctcaggat ---------- -----ccttt aatgtgatta atacgggt--
LECV CO22-    ttatttaatc atctcaggat ---------- -----tcttt aatgtgatta atacgggt--
LECV BA18-    ttatttaatc atctcaggat ---------- -----tctct aatgtgatta atacgggt--
LECV BA18-    ttatttaatc atctcaggat ---------- -----tctct aatgtgatta atacgggt--
LECV ER19-    ttatttaatc atctcaggat ---------- -----tcttt aatgtgatta atacgggt--
LECV 22819    ttatttaatc atctcaggat ---------- -----tctct aatgtgatta atacgggt--
```

```
BAV BA10-A   atatttaatc atctcaggac ---------- ------cttt aatgtgatta atgcgggc--
BAV BA16-L   atatttaatc atctcaggac ---------- ------cttt aatgtgatta atgcgggt--
BAV BA02-C   atatttaatc atctcaggac ---------- ------cttt aatgtgatta atgcgggt--
BAV BA17-S   atatttaatc atctcaggac ---------- ------cttt aatgtgatta atgcgggt--
BAV BA11-A   atatttaatc atctcaagac ---------- ------cttt aatgtaatta atgcgggt--
BAV BA02-C   atatttaatc atttcaggac ---------- ------cttt aatgtgatta atgcgggt--
BAV BA02-C   atatttaatc atttcaggac ---------- ------cttt aatgtgatta atgcgggt--
BAV BA02-C   atatttaatc atctcaggac ---------- ------cttt aatgtgatta atgcgggt--
BAV Hu3969   atatttaatc atctcaggac ---------- ------cttt aatgtgatta atgcgggt--
ANDV CHI-9   ata-ttaatc atctcaggac ct-------- ----atcctt aatgtgatta atagggtt--
ANDV AREB1   ata-ttaatc atctcagaat ct-------- ----atcctt aatgtgatta atagggtc--
ANDV Epuye   ata-ttaatc atctcaggat ct-------- ----atcctt aatgtgatta atagggtc--
ANDV Epili   ata-ttaatc atctcaggat ct-------- ----atcctt aatgtgatta atagggtc--
ANDV NRC-2   ata-ttaatc atctcaggac ct-------- ----atcctt aatgtgatta atagggtc--
ANDV NRC-4   ata-ttaatc atctcaggac ct-------- ----atcctt aatgtgatta atagggtc--
ANDV NRC-6   ata-ttaatc atctcaggat ct-------- ----atcctt aatgtgatta atagggtc--

             ....|....| ....|....| ....|....| ....|....| ....|....| ....|....|
                  1505       1515       1525       1535       1545       1555
SNV NM H10   -tag----ta ttgtattatg tattgttagt taagcttatt aaatcttgtt aagtttgtta
RIOMV HTN-   ttagtatcta tgaatgtatg ggtggtgggt ---------- -----aatgt catactactg
LANV 510B    ttagtatcta tgaatgtatg ggtggtgggt ---------- -----aatgt catactactg
CHOV 588     ---------- ttacaatgtg atgggtgggc aa-------- ----tgggtt tattgcacta
JABV AKM96   ---------- ---------- ---------- ---------- ---------- --ggtcatgt
MAPV HV-97   --------ct ttataatgtg atgggtgg-- ---------- -----gggtg gtgattacta
JUQV oln19   ---------- ttattataag ggtggtagat ta-------- ----ggggtg tttactatta
JUQV LH      ---------- ttattataag ggtggtagat ta-------- ----ggggtg tttatcatta
PERV 14403   ---------a ttattataag ggtggtgt-- ---------- -----tgggt cttattacta
MACV 13796   ---------- atattataag ggtgggaagt ---------- -----gggtt t--actacta
ORNV O1229   ---------- ttactatata aggggtgg-- ---------- -----tgggt tcaattacta
ORNV AND N   ---------- ttactatata aggggtgg-- ---------- -----tgggt tcaattacta
LECV BMJ-N   ---------- tgattatgag ggtggtgagt ---------- -----gggtt tcaattacta
LECV BMJ-O   ---------- tgatcatgag ggtggtaagt ---------- -----gggtt tcatttacta
LECV Plata   ---------- tgattataag ggtggtaagt ---------- -----gggtt tcaattacta
LECV CO22-   ---------- tgatcataag ggtggtaagt ---------- -----gggtt tcaattacta
LECV BA18-   ---------- tgattataag ggtggtaagt ---------- -----gggtt tcatttacta
LECV BA18-   ---------- tgattataag ggtggtaagt ---------- -----gggtt tcaattacta
LECV ER19-   ---------- tgattataag ggtggtaagt ---------- -----gggtt tcaattacta
LECV 22819   ---------- tgatcataag ggtggtaagt ---------- -----gggtt tcaattacta
BAV BA10-A   ---------- ttattataag ggtggtaagt ---------- -----gggtt tcaattacta
BAV BA16-L   ---------- ttattataag ggtggtaagt ---------- -----gggtt tcaattacta
BAV BA02-C   ---------- ttattataag ggtggtaagt ---------- -----gggtt tcaattacta
BAV BA17-S   ---------- ttattataag ggtggtaagt ---------- -----gggtt tcaattacta
BAV BA11-A   ---------- ttattataag ggtggtaagt ---------- -----gggtt tcaattacta
BAV BA02-C   ---------- ttattataag ggtggtaagt ---------- -----gggtt tcaattacta
BAV BA02-C   ---------- ttattataag ggtggtaagt ---------- -----gggtt tcaattacta
BAV BA02-C   ---------- ttattataag ggtggtaagt ---------- -----gggtt tcaattacta
BAV Hu3969   ---------- ttattataag ggtggtaagt ---------- -----gggtt tcaattacta
ANDV CHI-9   ---------- ttattataag ggcagttaa- ---------- -----tgggg ttggttacta
ANDV AREB1   ---------- ttattataag ggcggttaa- ---------- ------gggg ttagatacta
ANDV Epuye   ---------- ttattataag ggcggttaa- ---------- ------gggg tcagatacta
ANDV Epili   ---------- ttattataag ggcggttaa- ---------- ------gggg tcagatacta
ANDV NRC-2   ---------- ttattatagg gttggttaa- ---------- ------gggg tcagttacta
ANDV NRC-4   ---------- ttattataag ggcggttaa- ---------- ------gggg tcaattacta
ANDV NRC-6   ---------- ttattataag ggtggttaa- ---------- ------gggg tcagatacta

             ....|....| ....|....| ....|....| ....|....| ....|....| ....|....|
                  1565       1575       1585       1595       1605       1615
SNV NM H10   ag-------- -ttttgttaa gttgtattaa gttttgttaa gtttcggtaa gtctgttaaa
RIOMV HTN-   atgggaggat ttactgatac gggtaaagaa ---------- ---------- ----------
LANV 510B    atgggaggat ttactgatac gggtaaagaa ---------- ---------- ----------
CHOV 588     at-------- -------tta gggttaaggg ccataattga tttgtagtaa ttagataa--
JABV AKM96   at-------- -------tac gggtatgggg tggtttctta cttattgcac tagatatata
MAPV HV-97   a--------- ---------- gggtaaaggg ccacaatca- ---------- ----------
```

```
JUQV oln19   at-------- -------ccg ggtttatggg ccactactg- ---------- ----------
JUQV LH      at-------- -------ctg ggtttatggg ccactactg- ---------- ----------
PERV 14403   at-------- -------tac gggtaa---- --attacta- ---tttgcac tattttttaa
MACV 13796   at-------- -------tac gggt------ ----tactg- tcctttgcac tactatta--
ORNV O1229   ac-------- -------tac gggtaagggg acattaat-- --ctttgcac tagggtta-a
ORNV AND N   ac-------- -------tac gggtaagggg acattaat-- --ctttgcac tagggtta-a
LECV BMJ-N   ac-------- -------tac gggtagggga acactactg- tcctttgcac tagggtta-a
LECV BMJ-O   ac-------- -------aat gggtaaggga acactacta- tcttttgcac tagggtta-a
LECV Plata   ac-------- -------tac gggtagggga acactacta- tcctttgcac tagggtta-a
LECV CO22-   ac-------- -------tat gggtggggga acactacta- tcctttgcac tagggtta-a
LECV BA18-   ac-------- -------tgt gggtatggga acactacta- tcctttgcac tagggtta-a
LECV BA18-   ac-------- -------tgt gggtatggga acactacta- tcctttgcac tagggtta-a
LECV ER19-   ac-------- -------tgt gggtatggga acactacta- tcctttgcac tagggtta-a
LECV 22819   ac-------- -------ttt gggtatggga acactacta- tcctttgcac tagggtta-a
BAV BA10-A   ac-------- -------tac gggtaaggg- acactacta- tcctttgcac tggggtta-a
BAV BA16-L   ac-------- -------tac gggtaaggg- acactacta- tcctttgcac tggggtta-a
BAV BA02-C   ac-------- -------tac gggtaaggg- acactacta- tcctttgcac tggggtta-a
BAV BA17-S   ac-------- -------tac gggtaaggg- acactacta- tcctttgcac tggggtta-a
BAV BA11-A   ac-------- -------tac gggtaaggg- acactacta- tcctttgcac tggggtta-a
BAV BA02-C   ac-------- -------tac gggtaaggg- acactacta- tcctttgcac tggggtta-a
BAV BA02-C   ac-------- -------tac gggtaaggg- acactacta- tcctttgcac tggggtta-a
BAV BA02-C   ac-------- -------tac gggtaaggg- acactacta- tcctttgcac tggggtta-a
BAV Hu3969   ac-------- -------tac gggtaaggg- acactacta- tcctttgcac tggggtta-a
ANDV CHI-9   ac-------- -------tat gggtaagggt tcattacca- -ttttgcac tagggttaaa
ANDV AREB1   ac-------- -------tac gggtaagggt tcattactat cctttgcac tagggttaga
ANDV Epuye   ac-------- -------tac gggtaagggt tcattactat cctttgcac tagggttaaa
ANDV Epili   ac-------- -------tac gggtaagggt tcattactat cctttgcac tagggttaaa
ANDV NRC-2   ac-------- -------tac gagtaagggt tcattactan nttttgcac tagggttaga
ANDV NRC-4   ac-------- -------tac gggtaggggt tcattactan tttttgcac tagggttaga
ANDV NRC-6   ac-------- -------tac gggtaagggt tcattactat cctttgcac tagggttaaa

             ....|....| ....|....| ....|....| ....|....| ....|....| ....|....|
                 1625       1635       1645       1655       1665       1675
SNV NM H10   tg-----tag ttaatcttga tagtattaag tcatatgtat gggggtgtt tagt---gtt
RIOMV HTN-   --------tat cagggatttg tataac---- -aatcatcgt aaaagatatt tagt--ttat
LANV 510B    --------tat cagggatttg tataac---- -aatcatcgt aaaagatatt tagt--ttat
CHOV 588     ---------- gttagattta agtgatta-g ttaagtttaa gtaattagtt aagtttaggt
JABV AKM96   taactgtga- ttattatttg cactagta-c tgatgaatgt aagggtggtt t-----cgat
MAPV HV-97   acactatcat ctcatattta atcagt---- ---------- ---------- ---a---gat
JUQV oln19   ---------- ttcttatttg cactaa---- -ggggtgatg gtggggagtt att-----ac
JUQV LH      ---------- ttcttatttg cactaa---- -agggttaag gtggggagta att-----at
PERV 14403   taa------- -atatatttg cactac---- ---------- ---agatttt aa--------
MACV 13796   ---------- aatacatttg cactatt--- -ggggaaatg gggatataca tgta---gtc
ORNV O1229   gggtcattat cttgtagttg cactaca--- -ggggattgg gatat-ggtt t---------
ORNV AND N   gggtcattat cttatagttg cactaca--- -ggggatcgg gatatgggtt t---------
LECV BMJ-N   gggccattat tatatatttg cactac---- -tggggtagg gttaggtgtt a---------
LECV BMJ-O   gggccattat tctatatttg cactaa---- -tggggtaag gttaaatgtt a---------
LECV Plata   gggccattag tatatgtttg cactac---- -taggattag gggtaatgtt a---------
LECV CO22-   gggccattac tatatatttg cactac---- -tgggtttag ggttaatgtc a---------
LECV BA18-   gggccactat tatatatttg cactac---- -tgggattag ggttaatgtt a---------
LECV BA18-   gggccactat tatatatttg cactac---- -tggggttag ggttaatgtt a---------
LECV ER19-   gggccactat tatatatttg cactac---- -tgggattag ggttaatgtt a---------
LECV 22819   gggccactat tatatatttg cactac---- -tgggattag ggttaatgtt a---------
BAV BA10-A   gggccattat cttatatttg cactact--- -tggggttgg gataatgggt a---------
BAV BA16-L   gggtcattat cttatatttg cactact--- -tggggttgg gataatgggt a---------
BAV BA02-C   gggccattat cttatatttg cactact--- -tggggttgg gataatgggt a---------
BAV BA17-S   gggccattat cttatatttg cactact--- -tggggttgg gataatgggt a---------
BAV BA11-A   gggccattat cttatatttg cactact--- -tggggttgg gataatgggt a---------
BAV BA02-C   gggccattat cttatatttg cactact--- -tggggttgg gataatgggt a---------
BAV BA02-C   gggccattat cttatatttg cactact--- -tggggttgg gataatgggt a---------
BAV BA02-C   gggccattat cttatatttg cactact--- -tggggttgg gataatgggt a---------
BAV Hu3969   gggccattat cttatatttg cactact--- -taggttgg gataatgggt a---------
ANDV CHI-9   gggccactac attgtatttg cactaa---- -gggaaatgg gaggtgggtt agt-----tt
ANDV AREB1   gggccactac attgtatttg cactac---- -ggggaatgg gaggtgggtt agt-----ct
```

```
ANDV Epuye   gggccactac attgtatttg cactac---- -gggaaatgg gaggtgggtt agt-----ct
ANDV Epili   gggccactac attgtatttg cactac---- -gggaaatgg gaggtgggtt agt-----ct
ANDV NRC-2   gggccactac attgtatttg cactac---- -gggaaatgg gaggtgggtt agc-----tt
ANDV NRC-4   gggccactac atcgtatttg cactac---- -gggaaatgg gaggtgggtt agt-----ct
ANDV NRC-6   gggccactac attgtatttg cactac---- -gggaaatgg gaggtgggtt agt-----ct

             ....|....| ....|....| ....|....| ....|....| ....|....| ....|....|
                 1685       1695       1705       1715       1725       1735
SNV NM H10   aagtcattgt ttattgt-tt attgttatct --------gt ttaatgctaa gtctatatga
RIOMV HTN-   atattataga attcatg--- ---------- --------ag ttaattacta aactggttga
LANV 510B    atattataga attcatg--- ---------- --------ag ttaattacta aactggttga
CHOV 588     aattagttga ggttaag-tg atttaggtaa ----gttaag ataattaaga atat--ttag
JABV AKM96   atatcttagt ---------- ---------- ---------a gtaatcataa gttt-----g
MAPV HV-97   aagtaattgt tgttaag--- ---------- --------ac ataggtgtaa gttt------
JUQV oln19   atttattgtt tactaga-tc tta--tagat t--------g ataagtttag gtttaagtag
JUQV LH      atttactgtt tactata-tt atatgtagat t--------g ataagtttag gattaagtag
PERV 14403   atactttata tgttatc-cc attattgtaa tccatttaat caaattaggt gttt------
MACV 13796   agtatgtatt caatatg-ta ttcagtgtgt ---------- atcgttagtt gtttagttgt
ORNV O1229   acattataat ctatagg-tc aata------ ----tatagg ataggtataa gtatgtatag
ORNV AND N   acattataat ctatagg-tt aata------ ----tatagg ataggtataa gtatgtatag
LECV BMJ-N   agtccatag- ctttagg-ta attgataagt tagttataag atagatttag aaaa------
LECV BMJ-O   agtccttag- -tttaga-ta attgataagt tagttataag ataga-ttag aact------
LECV Plata   agtttatagt ttgtaat-ta gtcgataaga tagttataaa gtagacatag gaat------
LECV CO22-   agttcatag- tcccaat-ta gttgataaga tagtgataag atagacatag ggtt------
LECV BA18-   agtccatagt tttcagt-ta gccgataaga tagtgataag atagacatag gaat------
LECV BA18-   agtccatagt tttcaat-ta gccgataaga tagtgataag atagacatag gaat------
LECV ER19-   agttcatagt tttcaat-ta gccgataaga tagtgataag atagacatag gaat------
LECV 22819   agtccatag- tttcaag-ta gccgataaga tagtgataag atagacatag gaat------
BAV BA10-A   gaattatagt tcataag-tt agcattgtaa tagtcctagg ttaagtatag attt------
BAV BA16-L   gaattatagt ttataag-tt agcattataa tagtcctagg ttaagtatag gttt------
BAV BA02-C   gaattatagt tcataag-tt agcattataa tagtcctagg ttaagtatag gttt------
BAV BA17-S   gaattatagt tcataag-tt agcattctaa tagtcctagg ttaagtatag gttt------
BAV BA11-A   gaattatagt tcataag-tt agcattctaa tagtcctagg ttaagtatag gttt------
BAV BA02-C   gaattatagt tcataaa-tt agcattataa tagtcctagg ttaagtatag gttt------
BAV BA02-C   gaattatagt tcataaa-tt agcattataa tagtcctagg ttaagtatag gttt------
BAV BA02-C   gaattatagt tcataaa-tt agcattataa tagtcctagg ttaagtatag gttt------
BAV Hu3969   ggatcatagt tcataag-tt agcattgtga tagtcctagg ttaagtatag attt------
ANDV CHI-9   gtatt-tagt tgttaagttt ttta------ ---------- ---------- -------taa
ANDV AREB1   atattgtagt tgttaag-tt tttg------ ---------- ---------- -------tac
ANDV Epuye   atattgtagt tgttaag-tt tttg------ ---------- ---------- -------tac
ANDV Epili   atattgtagt tgttaag-tt tttg------ ---------- ---------- -------tac
ANDV NRC-2   atattatagt tgttaag-tt tttg------ ---------- ---------- -------tat
ANDV NRC-4   atattatagt tgttaag-tt tttg------ ---------- ---------- -------tat
ANDV NRC-6   atattgtatt tgttaag-tt tttg------ ---------- ---------- -------tac

             ....|....| ....|....| ....|....| ....|....| ....|....| ....|....|
                 1745       1755       1765       1775       1785       1795
SNV NM H10   t--ttattaa tcaaa---at aagatttata tacatagatt tattttatat acatagattt
RIOMV HTN-   ttat------ --------ga tttgatcata t--------- ---------- ----ggtgct
LANV 510B    ttat------ --------ga tttgatcata t--------- ---------- ----ggtgct
CHOV 588     ccgtatttac tcattag-gt ataagtcata tatat----- -------cag atgtggtaat
JABV AKM96   ttatagtcaa tcatt--tat gttagttatg tata------ ---------- -----tgaat
MAPV HV-97   tctttgatgt ttaat----- -----ctgta t--------- ---------- -----gtaat
JUQV oln19   ccataattaa ataggatcac actggctata t--------- ---------- -----aaaat
JUQV LH      ccataattat gtagaatcat actggctata t--------- ---------- -----gagat
PERV 14403   ---taagtaa gtcat---ta actagctgta t--------- ---------- -----gaaat
MACV 13796   t--taggtaa gtagc---ta actagttgta t--------- ---------- -----gaaat
ORNV O1229   tagtaagtat ataag---ta gatggccaaa t--------- ---------- -----gaaat
ORNV AND N   tagtaagtat ataag---ta gatggccaaa t--------- ---------- -----gaaat
LECV BMJ-N   ---tgattaa gtaga---ta aatagccaga t--------- ---------- -----gaagt
LECV BMJ-O   ---tgattaa gtgga---ta aatagccaga t--------- ---------- -----gacat
LECV Plata   ---tgattaa gtagg---ta aatagccaga t--------- ---------- -----gaaat
LECV CO22-   ---tgattaa gtaga---ta gatagccaga t--------- ---------- -----gaaat
LECV BA18-   ---tgattaa gtaga---ta gatagccaga t--------- ---------- -----gaaat
```

```
LECV BA18-   ---tgattaa gtaga---ta gatagccaga t--------- ---------- -----gaaat
LECV ER19-   ---tgattaa gtaga---ta gatagccaaa t--------- ---------- -----gaaat
LECV 22819   ---tgattaa gtaga---ta gatagccaga t--------- ---------- -----gaaat
BAV BA10-A   ---tgattag gtaag---ta attagccata t--------- ---------- -----gaaat
BAV BA16-L   ---tgattag gtaag---ca attagccata t--------- ---------- -----gaaat
BAV BA02-C   ---tgattag gtaag---ta attagccata t--------- ---------- -----gaaat
BAV BA17-S   ---taattag gtaag---ta attagccata t--------- ---------- -----gaaat
BAV BA11-A   ---tgattag gtaag---ta attagccata t--------- ---------- -----gaaat
BAV BA02-C   ---tgattag gtaag---ta attagccata t--------- ---------- -----gaaat
BAV BA02-C   ---tgattag gtaag---ta attagccata t--------- ---------- -----gaaat
BAV BA02-C   ---tgattag gtaag---ta attagccata t--------- ---------- -----gaaat
BAV Hu3969   ---tgattag gtaag---ta attagccata t--------- ---------- -----gaaat
ANDV CHI-9   tcatatgtta atgag---ga attagctata t--------- ---------- -----gatat
ANDV AREB1   tcataggttt ataag---ga atcagctata t--------- ---------- -----gagat
ANDV Epuye   tcataggttt ataag---ga atcagctata t--------- ---------- -----gagat
ANDV Epili   tcataggttt ataag---ga atcagctata t--------- ---------- -----gagat
ANDV NRC-2   tcataggtta atagg---ga atcagctata t--------- ---------- -----gaaat
ANDV NRC-4   tcataggttg ataag---ga atcagctata t--------- ---------- -----gaaat
ANDV NRC-6   tcataggttt ataag---ga atcagctata t--------- ---------- -----gagat

             ....|....| ....|....| ....|....| ....|....| ....|....| ....|....|
                  1805       1815       1825       1835       1845       1855
SNV NM H10   attttatcaa ctcattatca tctctcatca ttaatcaggg tt-tataaaa ttttctcagg
RIOMV HTN-   tgctgattga tcaatgagta a-acctgtca tttatcacta ctgtataatc attagacatg
LANV 510B    tgctgattga tcaatgagta a-acctgtca tttatcacta ctgtataatc attagacatg
CHOV 588     cttaggtagt ttctatgggc ------atca tagatcattg ggaaatgata atcaatgaga
JABV AKM96   cactgatata taa------- -------tta ttagttacat ---------- ----------
MAPV HV-97   ctttagttta t--gttaagt -------gtt ttgatcataa ---tgatgca tattgctgag
JUQV oln19   taattgttga ttggccggga ------ttgt tgtattct-- -----gtgta tgtaggttca
JUQV LH      taattgttga ttggccagaa ------ttat tatattaa-- -----gtgta tgtaggttta
PERV 14403   cagtgattga ccagctagg- -------tta ttaatta--- ---------- --taatca--
MACV 13796   caataatcga tcaact---- -------tag ttagtca--- -----ttata accaattata
ORNV O1229   cattgattga atggctagag ------tcaa tgta------ ---------- --tgatt---
ORNV AND N   cattgattga atggctagag ------tcaa tttat----- ---------- --tgatt---
LECV BMJ-N   cgttgattga ttggctattg -------ttg ttaatcacc- ----atagga tttagtt---
LECV BMJ-O   cattgattga tcggctattt -------tta ttaatcaat- ----atagga tttaatc---
LECV Plata   cattgattga ttggctatca -------ttg ttaatcat-- --------ag gattagt---
LECV CO22-   cattgattga ttggctatca -------atg ttaatcatt- ----atagga tttagtt---
LECV BA18-   cactgattga ttggctatca -------atg ttaatcatt- ----atagga tatagtt---
LECV BA18-   cactgattga ttggctatca -------atg ttaatcatt- ----atagga tatagtt---
LECV ER19-   cactgattga ttggctatca -------atg ttaatcatt- ----atagga tatagtt---
LECV 22819   cactgattga ttggctatca -------atg ttaatcatt- ----gtagga tatagtt---
BAV BA10-A   caatgattga ttggcttgca ------ttta ttgataat-- -----tagca tttagtt---
BAV BA16-L   caatgattga ttggcttgca ------ttta ttgataat-- -----tagca tttaatt---
BAV BA02-C   caatgattga ttggcttgca ------ttta ttgataat-- -----tagca tttaatt---
BAV BA17-S   caatgattga ttggcttgca ------ttta tcgataat-- -----tagca tttaatt---
BAV BA11-A   caatgattga ttggcttgta ------ttta tcgataat-- -----tagca tttaatt---
BAV BA02-C   caatgattga ttggcttgca ------ttta ttgataat-- -----tagca tttaatt---
BAV BA02-C   caatgattga ttggcttgca ------ttta ttgataat-- -----tagca tttaatt---
BAV BA02-C   caatgattga ttggcttgca ------ttta ttgataat-- -----tagca tttaatt---
BAV Hu3969   caatgattga ttggcttgca ------ttta ttgataat-- -----tagta tttagtt---
ANDV CHI-9   cactgattga ttggcta--- ------tttt taggttaagt ---aattgta gttaaata--
ANDV AREB1   cactgattga ttggctattt ------tttt taggttaagt ---gattaca tttaaata--
ANDV Epuye   cactgattga ttggctattt ------tttt taggttaagt ---gattaca tttaaata--
ANDV Epili   cactgattga ttggctantt ------tttt taggttaagt ---gattaca tttaaata--
ANDV NRC-2   cactgattga ttggctattt ------tttt taggttaagt ---gattgca tttaaata--
ANDV NRC-4   cactgattga ttggctattt ------tttt taggttaagt ---gattgca tttaaata--
ANDV NRC-6   cactgattga ttggctattt ------tttt taggttaagt ---gattaca tttaaata--

             ....|....| ....|....| ....|....| ....|....| ....|....| ....|....|
                  1865       1875       1885       1895       1905       1915
SNV NM H10   tctatcccta tattggaaga ttgttctatt aaccgt---- -------tca aactggaact
RIOMV HTN-   gt-------- ---------- ---------- -------aag ttttatgtgt aatcaattta
LANV 510B    gt-------- ---------- ---------- -------aag ttttatgtgt aatcaattta
```

```
CHOV 588     ctgactgct- --------ga tgggttgatt acctgtgatc tataaatttt tgttaatact
JABV AKM96   ---------- ---------- ---------- ---------- ---agtataa tgtttattat
MAPV HV-97   ttg------- ---------- -------atc aactgt-gta ttagaaattg tgttaatcat
JUQV oln19   t--------- ---------- ---------- ---------a tatactggta agttgactat
JUQV LH      t--------- ---------- ---------- ---------a tatattgata agtttactgt
PERV 14403   ---------- ---------- ---------- ---------- ------atta tgccagtata
MACV 13796   tc-------- ---------- ---------- --------at tatgccttta agtcaagtta
ORNV O1229   ---------- ---------- ---------- ---------- -------ata ggttaagtgt
ORNV AND N   ---------- ---------- ---------- ---------- -------ata ggttaagtgt
LECV BMJ-N   ---------- ---------- ---------- ---------- -------tta agttaagttt
LECV BMJ-O   ---------- ---------- ---------- ---------- -------tta agttaaggtt
LECV Plata   ---------- ---------- ---------- ---------- -------tta agttaaggtt
LECV CO22-   ---------- ---------- ---------- ---------- -------tta agttaaggtt
LECV BA18-   ---------- ---------- ---------- ---------- -------tta agttaaggtt
LECV BA18-   ---------- ---------- ---------- ---------- -------tta agttaaggtt
LECV ER19-   ---------- ---------- ---------- ---------- -------tta agttaaggtt
LECV 22819   ---------- ---------- ---------- ---------- -------tta agttaaggtt
BAV BA10-A   ---------- ---------- ---------- ---------- -------ata gattaagttc
BAV BA16-L   ---------- ---------- ---------- ---------- -------gta gattaagttt
BAV BA02-C   ---------- ---------- ---------- ---------- -------gta gattaagttt
BAV BA17-S   ---------- ---------- ---------- ---------- -------gta gattaagttt
BAV BA11-A   ---------- ---------- ---------- ---------- -------gta gattaagttt
BAV BA02-C   ---------- ---------- ---------- ---------- -------gta gattaagttt
BAV BA02-C   ---------- ---------- ---------- ---------- -------gta gattaagttt
BAV BA02-C   ---------- ---------- ---------- ---------- -------gta gattaagttt
BAV Hu3969   ---------- ---------- ---------- ---------- -------ata gattaagttc
ANDV CHI-9   ---------- ---------- ---------- ---------- ------gttg tgttaag-tt
ANDV AREB1   ---------- ---------- ---------- ---------- ------gttg tgttaagttt
ANDV Epuye   ---------- ---------- ---------- ---------- ------gttg tgttaagttt
ANDV Epili   ---------- ---------- ---------- ---------- ------gttg tgttaagttt
ANDV NRC-2   ---------- ---------- ---------- ---------- ------gttg ttttaagttt
ANDV NRC-4   ---------- ---------- ---------- ---------- ------gtta ttttaagttt
ANDV NRC-6   ---------- ---------- ---------- ---------- ------gttg tgttaagttt

             ....|....| ....|....| ....|....| ....|....| ....|....| ....|....|
                  1925       1935       1945       1955       1965       1975
SNV NM H10   aac------- ------actg tgactctgta a----agtcc agattatatt cacatctgta
RIOMV HTN-   aatttatgtt tgttcaactc t---atttta at---cattt ataactaata atcaggcata
LANV 510B    aatttatgtt tgttcaactc t---atttta at---cattt ataactaata atcaggcata
CHOV 588     gac-taatac taatcattat tatctcttta t-----aaat gatatcaaac taatgtttta
JABV AKM96   agttaagtaa gaa------- ----atttta a--------- ---------- ----------
MAPV HV-97   aat--atcaa taaccatat- ----atctta g--gtttagt taagtaagtt actaatt-ta
JUQV oln19   aatcaatgga taatcatata tcttgtatta gt----tttt aaggttacta acaaatcata
JUQV LH      aatcaatgga taatcatata tattttatta gt----tgtt aaggttacta acaaatcata
PERV 14403   atcatgtttg tgtt------ --taattata gtaataatcc tgaatatatt aacaat-ata
MACV 13796   aat------- ---------- ---------- -------cat tagatacact aatacttata
ORNV O1229   tac-tattta tgt------- ---attttta g-----gttt aagatctact aacaattatt
ORNV AND N   tac-tattta tgt------- ---actttta g-----gttt aagatctact aacaattatt
LECV BMJ-N   aat-cattag ga-------- ----ttctta g-----gttt aagataaact aacagttatt
LECV BMJ-O   aat-tatcag ta-------- ----tcctta g-----gttt aagataaact aacaattatt
LECV Plata   gat-cattag ta-------- ----ccctta g-----gttt aagataaact aacaattatt
LECV CO22-   aat-tattag ta-------- ----ttcttc at---ttttt aagataaact aacaattatt
LECV BA18-   aat-cattag ca-------- ----ttctta gt---ttttt aagataaact aacaattatt
LECV BA18-   aat-cattag ca-------- ----ttctta gt---ttttt aagataaact aacaattatt
LECV ER19-   aat-cattag ca-------- ----ttctta gt---ttttt aagataaact aacaattatt
LECV 22819   aat-cattag ca-------- ----ttctta gt---ttttt aagatgaact aacaattatt
BAV BA10-A   aat-tattaa tg-------- ----ttttta g------gtt aagattaact aacaatcatt
BAV BA16-L   aat-tgttaa tg-------- ----ttttta g------gtt aagattaact aacaatcatt
BAV BA02-C   aat-tgttaa tg-------- ----ttttta g------gtt aagattaact aacaatcatt
BAV BA17-S   aat-tgttaa tgt------- ----ttttta g------gtt aagattaact aacaatcatt
BAV BA11-A   aat-tgttaa tg-------- ----ttttta g------gtt aagattaact aacaatcatt
BAV BA02-C   aat-tgttaa tg-------- ----ttttta g------gtt aagattaact aacaatcatt
BAV BA02-C   aat-tgttaa tg-------- ----ttttta g------gtt aagattaact aacaatcatt
BAV BA02-C   aat-tgttaa tg-------- ----ttttta g------gtt aagattaact aacaatcatt
```

```
BAV Hu3969   aat-tgttaa tg-------- ----ttttta g------gtt aagattaact aacaatcatt
ANDV CHI-9   agtatgttaa gg-------- ----tttata g------gtt aagatttact aacaatcata
ANDV AREB1   agtatgttaa gg-------- ----cctata g------gtt aagatctact aacaattata
ANDV Epuye   agtgtgttaa gg-------- ----cctata g------gtt aagatctact aacaattata
ANDV Epili   agtgtgttaa gg-------- ----cctata g------gtt aagatctact aacaattata
ANDV NRC-2   agtatgttaa gg-------- ----cctata g------gtt aagatctact aacaattata
ANDV NRC-4   agtatgttaa gg-------- ----cctata g------gtt aagatctact aacaattata
ANDV NRC-6   agtgtgttaa gg-------- ----cctata g------gtt aagatctact aacaattata

             ....|....| ....|....| ....|....| ....|....| ....|....| ....|....|
                1985       1995       2005       2015       2025       2035
SNV NM H10   -----tatat ggatgtaaat ttcgttccgt taatagtaca tgacactaat gcttg-----
RIOMV HTN-   ---------tt agatgtaaat ttcattctca gctta----- ----------t gctttgca--
LANV 510B    ---------tt agatgtaaat ttcattctca gctta----- ----------t gctttgca--
CHOV 588     ttaatccatt ggatgtaaat ttcgttctg- ---------- -----gctat gcttt-----
JABV AKM96   -----ccttt agatgtaaat ttcgttctcc tgctt----- -----agctt gctttgca--
MAPV HV-97   aaactccaga agatgtaaat ttcgttccat gctt------ ------tgct gctttgctat
JUQV oln19   ctactcaaat agatgtacat ttcgttcaat gccttg---- -----ctact gcttc-----
JUQV LH      ctactcaaat agatgtaaat ttcgttcaat gccttg---- -----ctact gcttc-----
PERV 14403   ttataccaat agatgtaaat ttcattccat gctt------ ------tgct gcttt-----
MACV 13796   tcttatccat agatgtaaat ttcattccgt gctt------ ------tgct gcttt-----
ORNV O1229   gtatgccact agatgtaaat ttcgttcatt gctt------ ------tgct gcttt-----
ORNV AND N   gtatgccact agatgtaaat ttcgttcatt gctt------ ------tgct gcttt-----
LECV BMJ-N   ataatccatt ggatgtaaat ttcattcatt gctt------ ------tgct gcttt-----
LECV BMJ-O   gtaatccatt ggatgtaaat ttcgttcatt gctt------ ------tgct gcttt-----
LECV Plata   ataatccatt ggatgtaaat ttcgttcgtt gctt------ ------tgct gcttt-----
LECV CO22-   atactccatt ggatgtaaat ttcgttcatt gctt------ ------tgct gcttt-----
LECV BA18-   ataatccatt ggatgtaaat ttcgttcact gctt------ ------tgct gcttt-----
LECV BA18-   atactccatt ggatgtaaat ttcgttcatt gctt------ ------tgct gcttt-----
LECV ER19-   ataatccatt ggatgtaaat ttcgttcatt gctt------ ------tgct gcttt-----
LECV 22819   ataatccact ggatgtaaat ttcgttcatt gctt------ ------tgct gcttt-----
BAV BA10-A   -tactctatt ggatgtaaat ttcgttcatt gctt------ ------tgct gc-ta-----
BAV BA16-L   -tactctatt ggatgtaaat ttcgttcatt gctt------ ------tgct gc-tt-----
BAV BA02-C   -tactctatt ggatgtaaat ttcgttcatt gctt------ ------tgct gc-tt-----
BAV BA17-S   -tactctatt ggatgtaaat ttcgttcatt gctt------ ------tgct gc-tt-----
BAV BA11-A   -tactctatt ggatgtaaat ttcgttcatt gctt------ ------tgct gc-tt-----
BAV BA02-C   -tactctatt ggatgtaaat ttcgttcatt gctt------ ------tgct gc-tt-----
BAV BA02-C   -tactctatt ggatgtaaat ttcgttcatt gctt------ ------tgct gc-tt-----
BAV BA02-C   -tactctatt ggatgtaaat ttcgttcatt gctt------ ------tgct gc-tt-----
BAV Hu3969   -tactccatt ggatgtaaat ttcgttcatt gctt------ ------tgct gc-tt-----
ANDV CHI-9   ttatgtcatt agatgtaaat ttcattcctg gcttg----- -----cttct gc-tt-----
ANDV AREB1   ttatgtcatt ggatgtaaat ttcgttcctg gcttg----- -----cttct gc-tt-----
ANDV Epuye   ttatgtcatt ggatgtaaat ttcgttcctg gcttg----- -----cttct gc-tt-----
ANDV Epili   ttatgtcatt ggatgtaaat ttcattcctg gcttg----- -----cttct gc-tt-----
ANDV NRC-2   ttatgtcatt ggatgtaaat ttcgtttttg gcatg----- -----cttct gc-tt-----
ANDV NRC-4   ttatgtcatt ggatgtaaat ttcgttcttg gcttg----- -----cttct gc-tt-----
ANDV NRC-6   ttatgtcatt ggatgtaaat ttcgttcctg gcttg----- -----cttct gc-tt-----

             ....|....| ....|....| ....|....| ....|....| ....|....| ....|....|
                2045       2055       2065       2075       2085       2095
SNV NM H10   -ctttgctac taattgattt cttcattaca acaa-tctac ctcattgt-- -ccctatccc
RIOMV HTN-   gcactaacac taact----- ------tac- acatttctac ctcaaccact taccctcccc
LANV 510B    gcactaacac taact----- ------tac- acatttctac ctcaaccact taccctcccc
CHOV 588     -gcttaatac taacc----- -------aca acat-tctac ctcatcccaa cccctttccc
JABV AKM96   -----tctac taa------- ------caca acat-tctac ctcaacccca aaccttcacc
MAPV HV-97   gccttttac taaca----- -----tcaca acaa--ctac ctcatcccaa atcccctccc
JUQV oln19   attgctatac taaca----- -----ctaca acaacactac ctcatccaca -acccctccc
JUQV LH      attgctatac taaca----- -----tcaca acaacactac ctcatccaca -acccctccc
PERV 14403   -gcgctatat taacc----- -----ttaca acaaagctac ctcatccata -acccttccc
MACV 13796   -atgctatac taatc----- ------ataa acaaacctac ctcatccaca gacccttccc
ORNV O1229   -acgctatac taacc----- -------aca acaa-actac ctcatccaaa -aaccctccc
ORNV AND N   -atgctatac taacc----- -------aca acaa-actac ctcatccaaa -aaccctctc
LECV BMJ-N   -ttgctacac taacc----- ------aaca acaacactac ctcatcccaa aacccttccc
LECV BMJ-O   -ctgctacac taatt----- ------caca acaacactac ctcatcccaa aaccctcccc
```

```
LECV Plata   -ttgctacac taact----- ------caca acaaaactac ctcatccaca tacccctccc
LECV CO22-   -ctgctatat taact----- ------caca acaacactac ctcatccaca -aaccctccc
LECV BA18-   -ctgctatat taact----- ------caca acaacactac ctcatccaca -acccctccc
LECV BA18-   -ctgctatat taact----- ------caca acaacactac ctcatccaaa -aaccctccc
LECV ER19-   -ctgctatat taact----- ------taca acaacactac ctcatccaca -aaccctccc
LECV 22819   -ctgctatat taact----- ------caca acaacactac ctcatccaca -aaccctccc
BAV BA10-A   -ctgccacac gagcc----- ------taca acaaagctac cgcatccact -acgccaact
BAV BA16-L   -ctgctacac taacc----- ------taca acaaaactac ctcatccact -acccctccc
BAV BA02-C   -ctgctacac taacc----- ------taca acaaaactac ctcatccact -acccctccc
BAV BA17-S   -ctgctacac taacc----- ------taca acgaaactac ctcatccact -acccctccc
BAV BA11-A   -ctgctacac taacc----- ------taca acaaaactac ctcatccact -acccctccc
BAV BA02-C   -ctgctacac taacc----- ------taca acaaaactac ctcatccact -acccctccc
BAV BA02-C   -ctgctacac taacc----- ------taca acaaaactac ctcatccact -acccctccc
BAV BA02-C   -ctgctacac taacc----- ------taca acaaaactac ctcatccact -acccctccc
BAV Hu3969   -ctgctacac taacc----- ------taca acaaaactac ctcatccact -acccctccc
ANDV CHI-9   -tcgcattgc taacc----- ------taca acaagactac ctcacccact -acccctccc
ANDV AREB1   -tcgcattgc taacc----- ------taca acataactac ctcatccaca -aaccctccc
ANDV Epuye   -tcgcattgc taacc----- ------taca acataactac ctcatccaca -caccctccc
ANDV Epili   -ttgcattgc taacc----- ------taca acataactac ctcatccaca -aaccctccc
ANDV NRC-2   -tcgcattgc taacc----- ------taca acataactac ctcatccaca -aaccctccc
ANDV NRC-4   -tcgcattgc taatc----- ------taca acataactac ctcatccaca -aaccctccc
ANDV NRC-6   -tcgcattgc taacc----- ------taca acataactac ctcatccaca -aaccctctc

             ....|....| ....|....| ....|....| ....|....| ....|....| ....|....|
                 2105       2115       2125       2135       2145       2155
SNV NM H10   cttccctatt acctca---- acaaaactac ctcattataa gtaaattctt gattgctttt
RIOMV HTN-   ttt--atact acctca---- tttattctac ctc-ataaat tttctttctt gattgctttt
LANV 510B    ttt--atact acctca---- tttattctac ctc-ataaat tttctttctt gattgctttt
CHOV 588     ttta-aaact acctca---- attatactac ctc-attagt tttatctctt gattgctttt
JABV AKM96   ctttaaaact acctca---- tttattctac ctc-aatagt gtgttttctt gattgctttt
MAPV HV-97   cttt-atact acctca---- attatactac ctcataagtt --ttattctt gattgctttt
JUQV oln19   ctt---aact acctca---- acaa-actac ctcaaataat gcttttctt gattgctttt
JUQV LH      ctt---aact acctca---- acaatactac ctc-aataat gcttttctt gattgctttt
PERV 14403   ctt---ttct acctca---c aacatactac ctc-acattc gatttttctt gattgctttt
MACV 13796   tt-----tct acctca--aa aaaatactac ctc-gaatat gatttttctt gattgcttct
ORNV O1229   ctt---ttct acctcatctc aataaactac ctc-aatttt gatttttctt gattgctttt
ORNV AND N   ctt---ttct acctcatctc aataaactac ctc-aatttt gatttttctt gattgctttt
LECV BMJ-N   ttt---atct acctca---t ataaaactac ctc-aatttc gatttctctt gattgctttt
LECV BMJ-O   ttt---atct acctca---c ataaaactac ctc-aatttc gatttctctt gattgctttt
LECV Plata   cta---ttct acctcatctc aataaactac ctc-aatttt gatttttctt gattgctttt
LECV CO22-   ctt---ttct acctca---a ataaaactac ctc-aatttc gatttctctt gattgctttt
LECV BA18-   ctt---ttct acctca---a ataaaactac ctc-aatttc gatttctctt gattgctttt
LECV BA18-   ctt---ttct acctca---a ataaaactac ctc-aatttt gatttctctt gattgc----
LECV ER19-   ctt---ttct acctca---a ataaaactac ctc-aatttc gatttctctt gattgctttt
LECV 22819   ctt---ttct acctca---a ataaaactac ctc-aattct gatttctctt gattgctttt
BAV BA10-A   ctt---atca acctca---c ataagaatac ctc-aagtgc aatttcac-- ----------
BAV BA16-L   ctt---atct acctca---a ataaaactac ctc-aaattc gatttctctt gattgctttt
BAV BA02-C   ctt---atct acctca---a ataaaactac ctc-aaattc gatttctctt gctgtctc--
BAV BA17-S   ctt---atct acctca---a ataaaactac ctc-aaattc gatttctctt gattgcttga
BAV BA11-A   ctt---atct acctca---a ataaaactac ctc-aaattt gatttctctt gattgctttt
BAV BA02-C   ctt---atct acctca---a ataaaactac ctc-aaattc gatttctctt gattgctttt
BAV BA02-C   ctt---atct acctca---a ataaaactac ctc-aaattc gatttctctt gattgctttt
BAV BA02-C   ctt---atct acctca---a ataaaactac ctc-aaattc gatttctctt gattgctttt
BAV Hu3969   ctt---atct acctca---a ataaaactac ctc-acattc gatttctctt gattgctttt
ANDV CHI-9   cta---ttct acctca---a cacatactac ctc-acattt gatttttctt gattgctttt
ANDV AREB1   cta---ttct acctca---a cacatactac ctc-atattt gatttttctt gattgctttt
ANDV Epuye   cta---ttct acctca---a cacatactac ctc-atattt gatttttctt gattgctttt
ANDV Epili   cta---ttct acctca---a cacatactac ctc-atattt gatttttctt gattgctttt
ANDV NRC-2   cta---ttct acctca---a cacatactac ctc-atattt gatttttctt gattgctttt
ANDV NRC-4   cta---ttct acctca---a cacatactac ctc-atattt gatttttctt gattgctttt
ANDV NRC-6   cta---ttct acctca---a cacatactac ctc-atattt gatttttctt gattgctttt

             ....|....| ....|..
                 2165       2175
```

```
SNV NM H10   caaggagcat actacta
RIOMV HTN-   caaggagcat actacta
LANV 510B    caaggagcat actacta
CHOV 588     caaggagcat actacta
JABV AKM96   caaggagcat actacta
MAPV HV-97   caaggagcat actacta
JUQV oln19   caaggagcat actacta
JUQV LH      caaggagcat actacta
PERV 14403   caaggagcat actacta
MACV 13796   caaggagcat acta---
ORNV O1229   caaggagcat actacta
ORNV AND N   caaggagcat actacta
LECV BMJ-N   caaggagcat aca----
LECV BMJ-O   caaggagcat actacta
LECV Plata   caaggagcat actacta
LECV CO22-   caaggagcat actacta
LECV BA18-   c--------- -------
LECV BA18-   ---------- -------
LECV ER19-   caaggag--- -------
LECV 22819   caaggagcat actacta
BAV BA10-A   ---------- -------
BAV BA16-L   caagg----- -------
BAV BA02-C   ---------- -------
BAV BA17-S   ---------- -------
BAV BA11-A   ca-------- -------
BAV BA02-C   caaggagc-- -------
BAV BA02-C   caaggagc-- -------
BAV BA02-C   caaggagcat actacca
BAV Hu3969   caaggagcat actacta
ANDV CHI-9   caaggagcat actactt
ANDV AREB1   caaggagcat actacta
ANDV Epuye   ca-------- -------
ANDV Epili   caaggagcat acna---
ANDV NRC-2   caa------- -------
ANDV NRC-4   caaggagca- -------
ANDV NRC-6   caaggagc-- -------
```
